# Engineering Framework for Intelligent Vision Inspection Systems (EF-IVIS): A Systems Engineering Methodology

**DOI:** 10.3390/s26175432

**Published:** 2026-08-27

**Authors:** Jacek Rysiński, Daniel Jancarczyk

**Affiliations:** Faculty of Mechanical Engineering and Computer Science, University of Bielsko-Biala, Willowa 2, 43-300 Bielsko-Biala, Poland; jrysinski@ubb.edu.pl

**Keywords:** systems engineering, intelligent vision inspection, machine vision, industrial metrology, artificial intelligence, industrial integration, decision gates, smart manufacturing

## Abstract

Industrial vision inspection systems increasingly combine machine vision, industrial metrology, artificial intelligence, industrial communication, and manufacturing automation within integrated cyber-physical production environments. Although extensive research addresses individual technologies, comparatively limited methodological support is available for coordinating engineering decisions across the complete development lifecycle of such systems. This paper presents the Engineering Framework for Intelligent Vision Inspection Systems (EF-IVIS), a domain-specific systems engineering approach organized into seven interconnected engineering domains: inspection requirements, image acquisition, illumination engineering, measurement strategy, artificial intelligence, industrial integration, and validation and continuous improvement. EF-IVIS was developed through a bottom-up synthesis of established engineering principles and four complementary industrial investigations addressing AI-based defect detection, measurement strategies, illumination conditions, and PLC-supported integration. The empirical assessment distinguishes direct experimental evidence, direct industrial evidence, cross-cutting retrospective evidence, and methodological support. Direct evidence supports selected aspects of image acquisition and the illumination engineering, measurement strategy, artificial intelligence, and industrial integration domains, whereas inspection requirements and validation and continuous improvement are supported primarily through cross-study synthesis and established systems engineering principles. The framework introduces cross-domain dependencies, application-specific Decision Gates, and feedback mechanisms for iterative engineering assessment. A worked industrial-integration example demonstrates how reported technical evidence can be translated into Decision Gate criteria without treating the reported values as universal thresholds. The present study supports the internal consistency and practical relevance of EF-IVIS, while its usability, repeatability, transferability, and complete lifecycle effectiveness require independent prospective validation.

## 1. Introduction

Industrial manufacturing has entered an era in which competitive advantage is determined not only by production efficiency but increasingly by the capability of manufacturing systems to generate, process, and utilize information throughout the entire production lifecycle. Digital transformation, mass customization, shortening product lifecycles, and increasing quality expectations have fundamentally changed the philosophy of production engineering. Modern manufacturing systems are no longer designed solely to produce components within specified tolerances but to continuously evaluate their own performance, detect process deviations, and support autonomous engineering decisions before non-conformities propagate through the production system [1,2,3,4,5,6,7,8].

Within this context, quality inspection has undergone a profound conceptual transformation. Traditionally, inspection represented the final stage of manufacturing, where products were classified as acceptable or defective after production had already been completed. Such an approach was appropriate for conventional manufacturing characterized by relatively stable production conditions, limited product variability, and clearly defined inspection procedures. Contemporary manufacturing environments, however, require a fundamentally different philosophy. Inspection systems are now expected to operate continuously, interact with production equipment, provide feedback to process control systems, and support engineering decisions throughout the complete manufacturing process rather than merely verifying product conformity [2,3,4,5,6,9,10,11,12,13].

**Figure 1:**
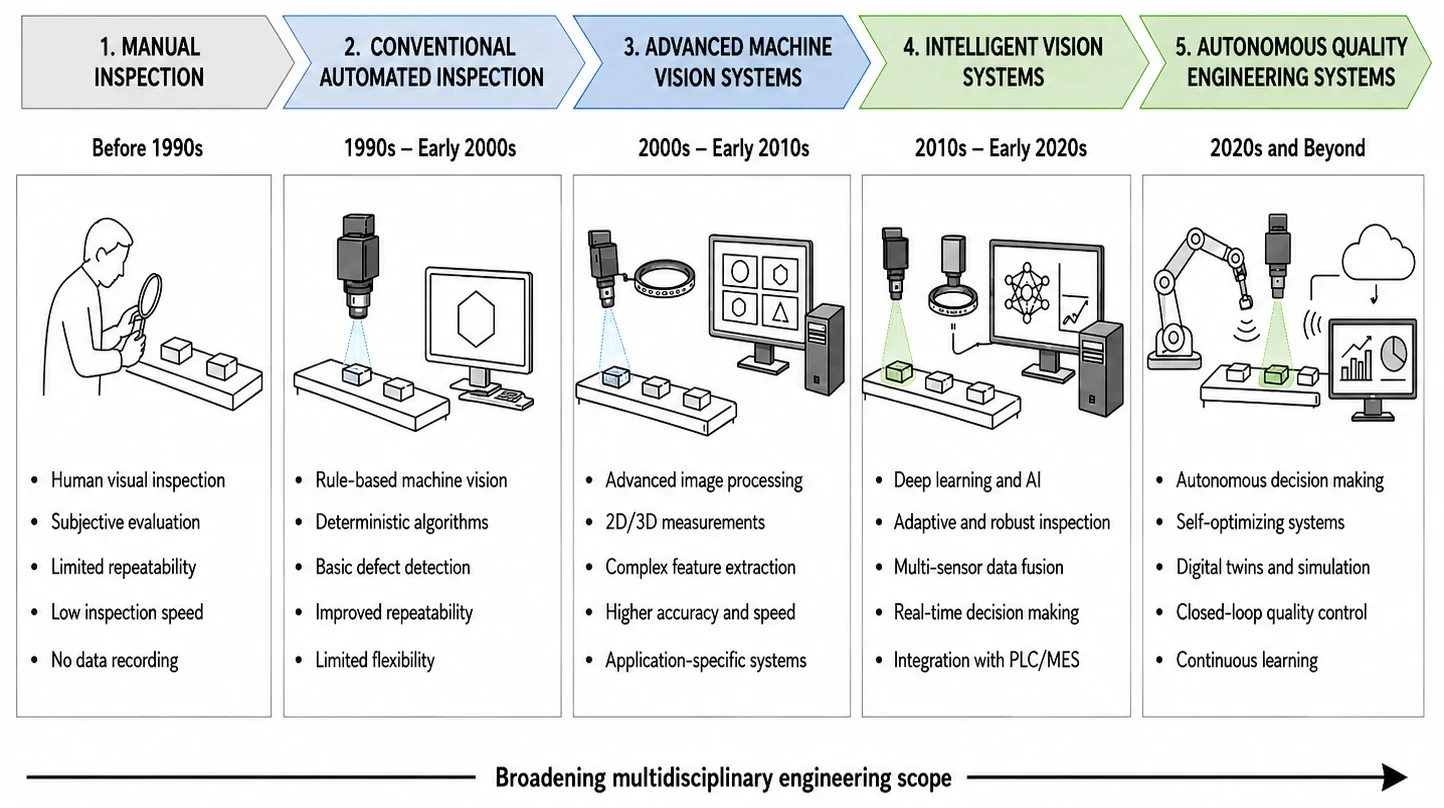
Evolution of engineering complexity in industrial quality inspection.

The evolution of industrial quality inspection is therefore not only a consequence of advances in sensing technologies or artificial intelligence but primarily the result of increasing engineering complexity within modern manufacturing systems. As production environments become increasingly interconnected, quality inspection is gradually evolving from a measurement activity into a multidisciplinary engineering function integrating optical sensing, image acquisition, artificial intelligence, industrial communication, automation, production control, and decision support [1,2,3,4,5,6,9,10,11,14,15,16,17,18,19,20,21,22,23,24,25]. The conceptual evolution of this increasing engineering complexity is summarized in Figure 1.

As shown in Figure 1, industrial quality inspection has progressively developed from manual inspection toward intelligent and increasingly autonomous engineering systems. This broader transformation provides the industrial context for interpreting contemporary vision inspection systems as cyber-physical engineering systems rather than isolated measurement devices.

In the context of intelligent vision inspection, the cyber-physical character of the system results from the closed interaction between physical manufacturing processes and computational decision-making. Physical products and process conditions are observed through optical sensing, the acquired information is transformed into measurements or classification decisions, and these decisions are communicated to production-control systems that may subsequently influence the physical process. The resulting sensing–computation–communication–control feedback loop is consistent with the general concept of cyber-physical production systems, in which computational and communication components are tightly integrated with physical manufacturing processes [2,3,4,5,6,7,8]. EF-IVIS addresses this interaction from an engineering perspective by explicitly linking sensing, measurement, artificial intelligence, industrial communication, control integration, validation, and lifecycle feedback within a common development structure.

Against this background, intelligent vision inspection systems operate across several interacting engineering domains. They may combine optical hardware, illumination systems, digital imaging, measurement algorithms, artificial intelligence, programmable logic controllers, industrial communication protocols, and manufacturing information systems within a common operational architecture [1,2,3,4,5,6,20,21,22,23,24,25,26,27,28,29,30,31,32,33]. Consequently, their development extends beyond the selection of individual hardware components or image-processing algorithms. Decisions made within one engineering domain may constrain subsequent design choices and influence system-level performance, creating dependencies that must be considered throughout system development [14,15,16,17,18,19].

Scientific research has extensively addressed individual technological aspects of machine vision. Significant progress has been achieved in image acquisition, optical metrology, deep learning, defect detection, semantic segmentation, anomaly detection, edge computing, explainable artificial intelligence, and industrial communication [26,27,28,29,30,31,32,33,34,35,36,37,38,39,40,41,42]. These developments have expanded the technical capabilities of industrial vision systems and increased the range of inspection tasks that can be addressed using automated and data-driven methods.

Industrial implementation, however, introduces engineering constraints that extend beyond laboratory evaluation of individual algorithms or components [1,2,3,4,5,6,9,10,11,14,15,16,17,18,19]. Image acquisition performance depends on factors including illumination conditions, optical configuration, sensor characteristics, environmental conditions, and product presentation [26,27,28,29,30,31]. AI-based inspection additionally depends on the characteristics and preparation of image data, model configuration, computational resources, and the selected decision strategy [32,33,34,35,36,37,38,39,40,41,42]. Deployment within a manufacturing environment may also introduce requirements associated with communication timing, synchronization with programmable controllers, result traceability, maintainability, operational reliability, and integration with surrounding production systems [20,21,22,23,24,25].

Inspection performance should therefore be considered at the system level rather than evaluated solely through the performance of an individual algorithm or hardware component [1,2,3,4,5,6,9,10,11,14,15,16,17,18,19]. Domain-specific indicators such as classification accuracy, detection performance, processing time, measurement repeatability, or communication latency remain essential, but industrial implementation additionally requires consistency among the engineering decisions that determine how these functions interact within the complete inspection architecture.

This systems-oriented perspective changes the engineering focus from identifying the individually best-performing camera, model, or measurement algorithm toward determining whether the selected technologies are mutually compatible and appropriate for the intended inspection task and production environment. Their selection, integration, verification, and subsequent validation must therefore be considered in relation to inspection requirements, upstream and downstream engineering decisions, and application-specific production constraints [14,15,16,17,18,19].

**Figure 2:**
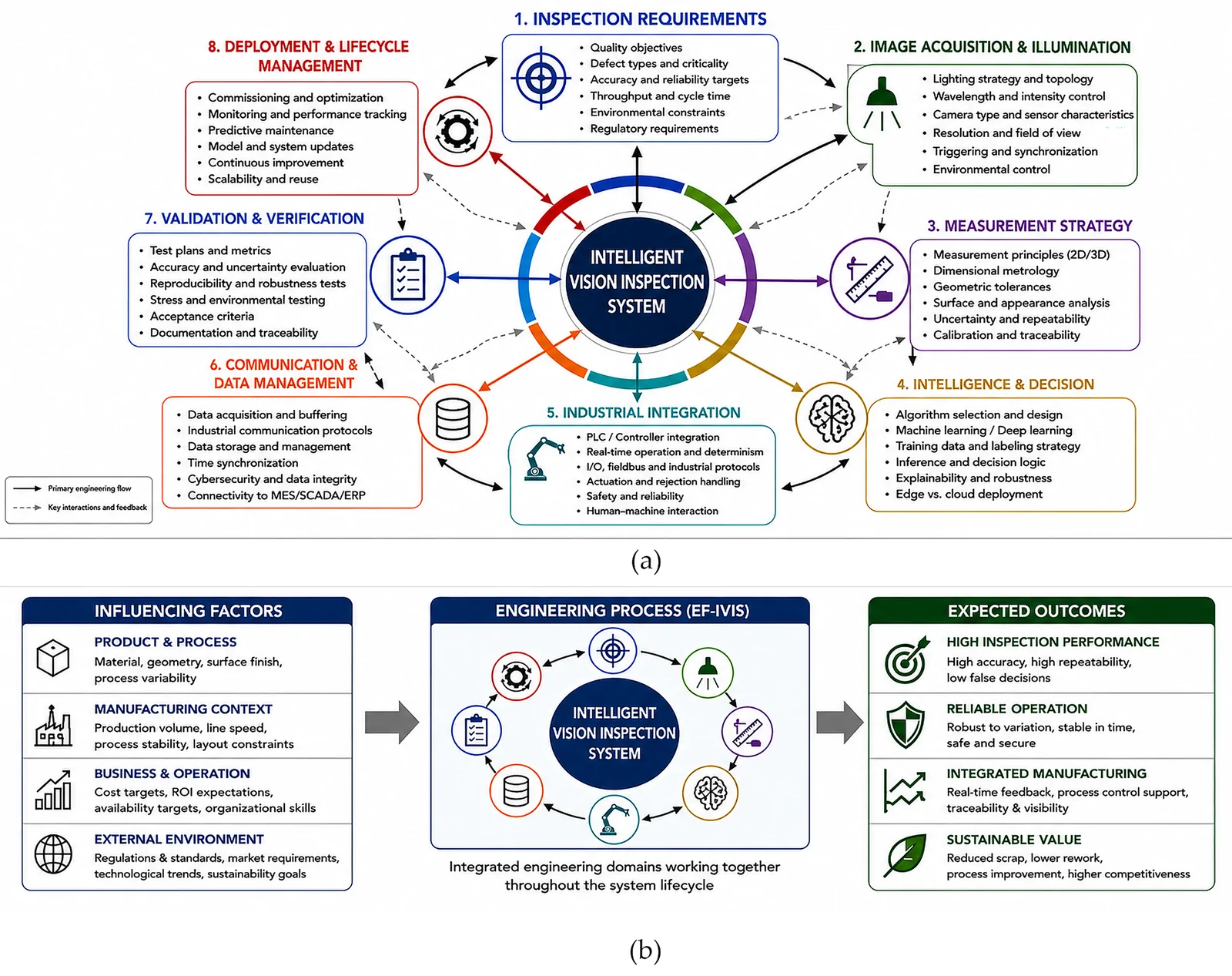
Engineering Decision Space for intelligent vision inspection systems: (**a**) core engineering domains and their interactions; (**b**) influencing factors and expected system-level outcomes.

The multidimensional character of these engineering decisions is illustrated in Figure 2 through the Engineering Decision Space adopted in this work. Rather than representing a specific hardware architecture, Figure 2 summarizes the principal technological and engineering areas that must be considered when developing an intelligent vision inspection system and highlights the dependencies that motivate their coordinated treatment within EF-IVIS.

The Engineering Decision Space identifies the fundamental relationships between inspection objectives, image acquisition, illumination strategy, measurement methodology, artificial intelligence, communication infrastructure, industrial automation, validation procedures, and operational deployment. Rather than treating these engineering domains independently, the proposed perspective assumes that every design decision propagates throughout the complete inspection architecture, influencing subsequent engineering activities as well as the final operational performance of the system [1,2,3,4,5,6,9,10,11,14,15,16,17,18,19].

Although numerous publications investigate individual technological components of intelligent inspection systems, comparatively limited attention has been devoted to methodologies supporting engineering decision-making during complete system development [26,27,28,29,30,31,32,33,34,35,36,37,38,39,40,41,42]. Existing studies predominantly optimize individual subsystems while assuming that the remaining components remain unchanged. However, industrial implementation consistently demonstrates that modifications introduced within one engineering domain frequently require corresponding adaptations across multiple remaining engineering domains of the inspection architecture [1,2,3,4,5,6,9,10,11,20,21,22,23,24,25]. As system complexity continues to increase, engineering experience remains one of the primary factors determining successful industrial deployment [14,15,16,17,18,19].

This observation reveals an important research gap. The current scientific literature provides extensive knowledge concerning individual technologies but offers comparatively limited support for engineers responsible for integrating these technologies into coherent industrial inspection systems [1,2,3,4,5,6,9,10,11,14,15,16,17,18,19,26,27,28,29,30,31,32,33,34,35,36,37,38,39,40,41,42]. Consequently, the development of intelligent vision inspection systems still relies predominantly on empirical engineering practice rather than structured design methodologies supported by experimental evidence.

The evolution of industrial vision inspection technologies and the corresponding expansion of engineering scope are summarized conceptually in Table 1 [1,2,3,4,5,6,9,10,11]. The table highlights the transition from isolated inspection activities toward increasingly integrated sensing, measurement, decision-making, automation, communication, and lifecycle functions.

Contributions of this Study

Based on the identified research gap, this study proposes the Engineering Framework for Intelligent Vision Inspection Systems (EF-IVIS), a domain-specific systems engineering framework for the systematic development of intelligent industrial vision inspection systems. EF-IVIS does not introduce new fundamental systems engineering principles. Instead, it operationalizes established systems engineering concepts for the specific requirements of intelligent vision inspection by integrating machine vision, industrial metrology, artificial intelligence, industrial automation, and manufacturing-system integration within a unified lifecycle-oriented engineering structure. The distinctive contribution of EF-IVIS lies in the explicit organization of these disciplines into interconnected engineering domains, the formalization of cross-domain dependencies, and the introduction of dedicated Decision Gates linking technical evidence with successive engineering decisions. The principal contributions of this work are summarized as follows:

1. A domain-specific Engineering Framework for Intelligent Vision Inspection Systems (EF-IVIS) is formulated, organizing the development lifecycle into seven interconnected engineering domains: inspection requirements, image acquisition, illumination engineering, measurement strategy, artificial intelligence, industrial integration, and validation and continuous improvement.
2. Cross-domain dependencies between these domains are explicitly identified and organized within a unified engineering structure, including the influence of image acquisition and illumination on measurement reliability and AI performance, as well as the constraints imposed by industrial integration on computation, communication, and deployment.
3. Industrial metrology, artificial intelligence, and automation are integrated within a common systems engineering process, allowing measurement uncertainty, data-driven decision-making, and production-system constraints to be considered jointly rather than as separate technological activities.
4. A sequence of Decision Gates is introduced to provide explicit engineering assessment points between successive development stages and to support traceable progression from inspection requirements to industrial deployment and lifecycle operation.
5. The proposed domain structure and cross-domain relationships are grounded in a synthesis of established systems engineering principles and four complementary industrial investigations covering AI-based defect detection, measurement methodology, illumination engineering, and PLC-supported industrial integration. The investigations provide direct experimental evidence for selected technological domains and retrospective empirical support for the overall framework structure.

Collectively, these contributions position EF-IVIS as a domain-specific systems engineering framework intended to bridge established systems engineering principles with the technological and operational requirements of intelligent industrial vision inspection. Its broader transferability and effectiveness across different development environments require prospective evaluation.

## 2. Engineering Research Methodology

### 2.1. Engineering Philosophy

The Engineering Framework for Intelligent Vision Inspection Systems (EF-IVIS) proposed in this study originates from the observation that the design of industrial vision inspection systems has evolved into a multidisciplinary engineering task requiring coordinated decision-making across multiple technological domains [1,2,3,4,5,6,9,10,11,14,15,16,17,18,19]. Unlike conventional studies that primarily investigate individual algorithms or hardware components [26,27,28,29,30,31,32,33,34,35,36,37,38,39,40,41,42], the present work adopts a systems engineering perspective in which inspection performance is considered to depend on interactions among multiple engineering decisions and technological domains [14,15,16,17,18,19].

The adopted methodology assumes that industrial implementation should not be approached solely through independent optimization of isolated subsystems. Instead, decisions concerning inspection objectives, image acquisition, illumination, measurement strategy, artificial intelligence, industrial communication, production integration, and validation should be considered as mutually dependent elements of a coordinated engineering process [1,2,3,4,5,6,9,10,11,14,15,16,17,18,19,20,21,22,23,24,25].

Consequently, EF-IVIS was developed using a bottom-up synthesis in which engineering knowledge obtained from complementary industrial investigations [43,44,45,46] was combined with established systems engineering principles, relevant standards, the scientific literature, and industrial engineering knowledge. The four investigations provided domain-specific experimental and industrial observations concerning artificial intelligence-based defect detection, measurement strategies, illumination engineering, and PLC-supported industrial integration. These observations were subsequently analysed together with methodological foundations from systems engineering and related engineering disciplines to identify recurring engineering activities, decision criteria, cross-domain dependencies, and feedback relationships.

Rather than constructing a theoretical framework first and subsequently validating it through the same experimental programme, the investigations [43,44,45,46] served primarily as sources of engineering knowledge during framework development and as retrospective evidence for assessing the relevance and internal consistency of the resulting domain structure. The current evidence therefore supports selected technological domains and cross-domain relationships, while prospective validation of EF-IVIS as a complete systems engineering framework remains a subject for future research.

**Figure 3:**
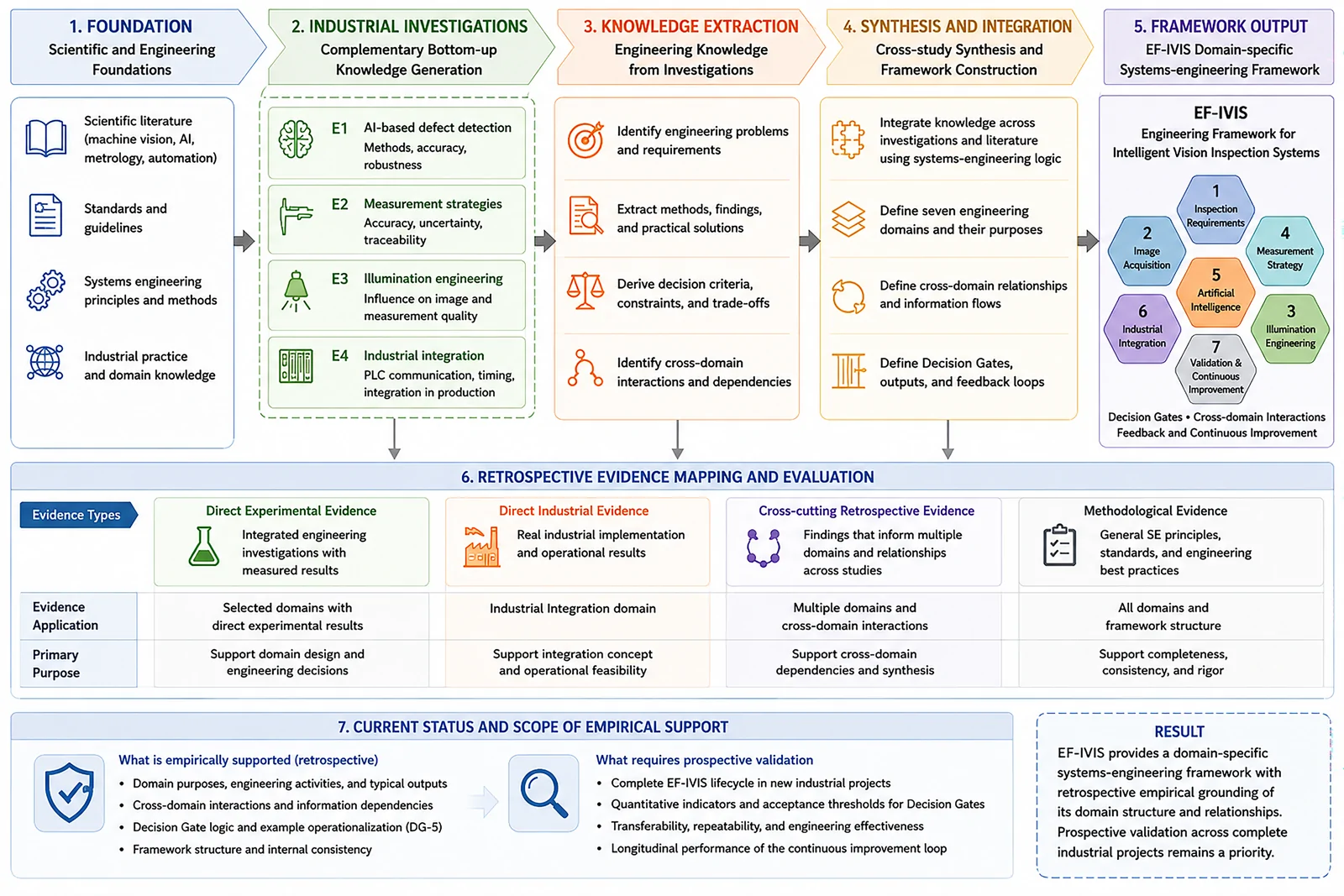
Development of the EF-IVIS framework from engineering knowledge sources to retrospective evidence mapping. The industrial investigations comprise AI-based defect detection [43], measurement strategies [44], illumination engineering [45], and industrial integration [46].

The overall development philosophy, knowledge sources, synthesis process, and retrospective evidence-mapping strategy are summarized in Figure 3.

### 2.2. Engineering Decision Process

The proposed methodology considers the development of an intelligent vision inspection system as a coordinated sequence of engineering decisions rather than primarily as a sequence of hardware or software selections [14,15,16,17,18,19]. Decisions made within one engineering domain may influence subsequent activities, constrain available design alternatives, and affect system-level performance [1,2,3,4,5,6,9,10,11].

The engineering decision process adopted within EF-IVIS is organized into seven principal domains:

- Inspection requirements;
- Image acquisition;
- Illumination engineering;
- Measurement strategy;
- Artificial intelligence;
- Industrial integration;
- Validation and continuous improvement.

These domains correspond to the principal areas of engineering decision-making identified within the Engineering Decision Space presented in Figure 2. Although the domains are presented in a logical development sequence, EF-IVIS does not assume a strictly linear process. Information generated during later stages may require revision of earlier engineering decisions, and operational feedback may initiate further iteration across one or more domains [7,8,14,15,16,17,18,19].

### 2.3. Experimental Research Programme

The engineering methodology proposed in this paper was developed using four complementary industrial investigations. Rather than representing independent validation studies, these investigations were interpreted as complementary sources of engineering knowledge supporting selected EF-IVIS domains [43,44,45,46]. Each investigation primarily addressed one or more engineering areas relevant to the development of intelligent industrial vision inspection systems.

The first investigation examined artificial intelligence-based defect detection, including dataset preparation, ROI definition and masking, model configuration, defect-area scoring, and Pass/Fail classification [43].

The second investigation focused on measurement and inspection strategies implemented within an industrial vision system. The study compared deterministic user-configured functions with AI-assisted tools in terms of inspection output, configuration characteristics, processing time, and sensitivity to object displacement and rotation. The results support consideration of deterministic and AI-assisted methods as complementary approaches, where their capabilities provide different types of inspection information [44,47,48,49,50].

The third investigation addressed illumination engineering and selected image acquisition conditions. Shape and colour recognition were examined under white, red, green, and blue illumination using fixed imaging geometry. The reported outcomes showed illumination-dependent changes in detection and classification, providing direct experimental support for illumination engineering and supporting evidence for selected image acquisition dependencies [26,27,28,29,30,31,45].

The fourth investigation addressed industrial integration by connecting machine vision inspection with a programmable logic controller through industrial communication. The study provided industrial evidence concerning camera–PLC communication, controller-level verification, timing, throughput, and selected operating perturbations [1,2,3,4,5,6,9,10,11,20,21,22,23,24,25,46]. The engineering scope of the coordinated research programme is summarized in Table 2.

### 2.4. Knowledge Integration Methodology

Unlike conventional review studies, EF-IVIS was not developed through literature synthesis alone. Instead, the framework integrates engineering knowledge derived from four successive industrial investigations [43,44,45,46] with established principles from systems engineering and the relevant technological domains [14,15,16,17,18,19]. Each investigation contributed experimental observations and engineering findings concerning one or more decision domains. These findings were subsequently generalized and organized into a hierarchical framework describing relationships between inspection requirements, technological decisions, validation activities, and industrial implementation. The resulting evidence integration and framework evaluation strategy is described in Section 2.5 and presented in detail in Section 4.

### 2.5. Evidence Integration and Framework Evaluation Strategy

To assess the practical relevance and internal consistency of EF-IVIS, observations obtained from four complementary industrial investigations were mapped onto the engineering domains of the proposed framework [43,44,45,46]. The investigations addressed AI-based defect detection, industrial measurement methodology, image acquisition and illumination engineering, and PLC-supported industrial integration. Together, they provide an empirical basis for examining whether the proposed EF-IVIS structure reflects engineering activities encountered during the development and implementation of intelligent vision inspection systems.

The evaluation distinguishes four complementary forms of evidence: direct experimental evidence, direct industrial evidence, cross-cutting retrospective evidence, and methodological support. Direct experimental evidence is assigned when a technological variable, configuration, or inspection function was explicitly examined under experimental conditions. Direct industrial evidence is assigned when engineering implementation or system behaviour was evaluated within an integrated industrial or production-control architecture. Cross-cutting retrospective evidence is derived from recurring observations identified across multiple investigations, whereas methodological support is derived from established systems engineering, requirements engineering, quality management, and intelligent manufacturing principles [1,2,3,4,5,6,7,8,9,10,11,12,13,14,15,16,17,18,19,51,52].

This distinction is necessary because the four investigations served both as sources of engineering knowledge during the bottom-up development of EF-IVIS and as retrospective evidence supporting its domain structure and cross-domain relationships [43,44,45,46]. Consequently, the present evaluation addresses the empirical grounding, internal consistency, and practical relevance of the framework, but it should not be interpreted as independent prospective validation of EF-IVIS throughout the complete lifecycle of a newly developed inspection system.

The detailed evidence-mapping criteria and the assignment of individual investigations to EF-IVIS domains are presented in Section 4.2.

### 2.6. Transition to the Engineering Framework

The coordinated research programme described above provides an empirical and methodological basis for the proposed Engineering Framework for Intelligent Vision Inspection Systems. Rather than representing isolated technological achievements, the individual investigations collectively establish the engineering knowledge required for systematic development of intelligent inspection systems [43,44,45,46].

The following section presents the proposed EF-IVIS framework and describes how engineering knowledge derived from the literature and industrial investigations [43,44,45,46] was organized into a unified domain-specific systems engineering structure supporting the design, implementation, assessment, deployment, and lifecycle improvement of intelligent vision inspection systems [1,2,3,4,5,6,9,10,11,14,15,16,17,18,19].

## 3. Engineering Framework Development

### 3.1. Framework Development Rationale

The increasing complexity of industrial vision inspection systems has changed the engineering process required for their systematic implementation. Contemporary inspection systems may integrate optical sensing, image acquisition, industrial metrology, artificial intelligence, industrial communication, programmable control, and production-management functions within interconnected cyber-physical environments [1,2,3,4,5,6,9,10,11,14,15,16,17,18,19,20,21,22,23,24,25]. Consequently, their development cannot be reduced to the independent selection or optimization of individual hardware and software components.

The existing scientific literature provides extensive knowledge concerning individual technological domains, including optical inspection, machine vision, industrial metrology, deep learning, industrial communication, and production automation [1,2,3,4,5,6,9,10,11,20,21,22,23,24,25,26,27,28,29,30,31,32,33,34,35,36,37,38,39,40,41,42]. However, these areas are commonly addressed through domain-specific methods and performance criteria, whereas industrial implementation requires coordinated decisions across several interacting engineering disciplines [14,15,16,17,18,19].

EF-IVIS was developed to address this domain-integration problem from a systems engineering perspective. The framework does not introduce new fundamental systems engineering principles or prescribe a new technological solution. Instead, it provides a domain-specific engineering structure that combines established systems engineering concepts with knowledge obtained from machine vision, industrial metrology, artificial intelligence, industrial automation, relevant standards, and four complementary industrial investigations [14,15,16,17,18,19,43,44,45,46].

The industrial investigations provided direct experimental or industrial evidence for selected technological areas, including illumination engineering, measurement strategy, artificial intelligence, and industrial integration, together with supporting observations relevant to image acquisition [43,44,45,46]. These findings were synthesized with established engineering principles to define the seven EF-IVIS domains, their principal activities and outputs, cross-domain dependencies, Decision Gates, and feedback mechanisms.

Accordingly, the framework development process comprised three principal methodological activities:

- Identification and structuring of the engineering domains relevant to intelligent vision inspection based on the scientific literature, standards, and systems engineering principles;
- Extraction and synthesis of experimentally or industrially supported engineering knowledge from the four complementary investigations [43,44,45,46];
- Integration of this knowledge into a unified domain-specific systems engineering framework with explicit dependencies, assessment points, and lifecycle feedback.

The resulting framework should therefore be interpreted as a structured engineering decision-support approach whose domain structure is informed by industrial evidence and established engineering principles. The current investigations provide retrospective empirical support for selected domains and cross-domain relationships, while prospective validation of EF-IVIS throughout the complete lifecycle of a newly developed industrial inspection system remains required.

### 3.2. Systems Engineering Architecture of EF-IVIS

The proposed framework follows the general philosophy of systems engineering, according to which complex engineering systems should be developed through a sequence of coordinated engineering activities rather than isolated technological decisions [14,15,16,17,18,19]. Accordingly, EF-IVIS organizes the development process into successive engineering domains that correspond to the natural lifecycle of industrial vision inspection systems.

Instead of prescribing specific cameras, illumination systems, neural networks, or industrial controllers, the framework defines engineering activities, engineering decisions, expected outputs, and validation criteria required during system development [14,15,16,17,18,19,20,21,22,23,24,25]. The resulting engineering architecture is organized into seven interacting engineering domains:

- Inspection requirements;
- Image acquisition;
- Illumination engineering;
- Measurement strategy;
- Artificial intelligence;
- Industrial integration;
- Validation and continuous improvement.

**Figure 4:**
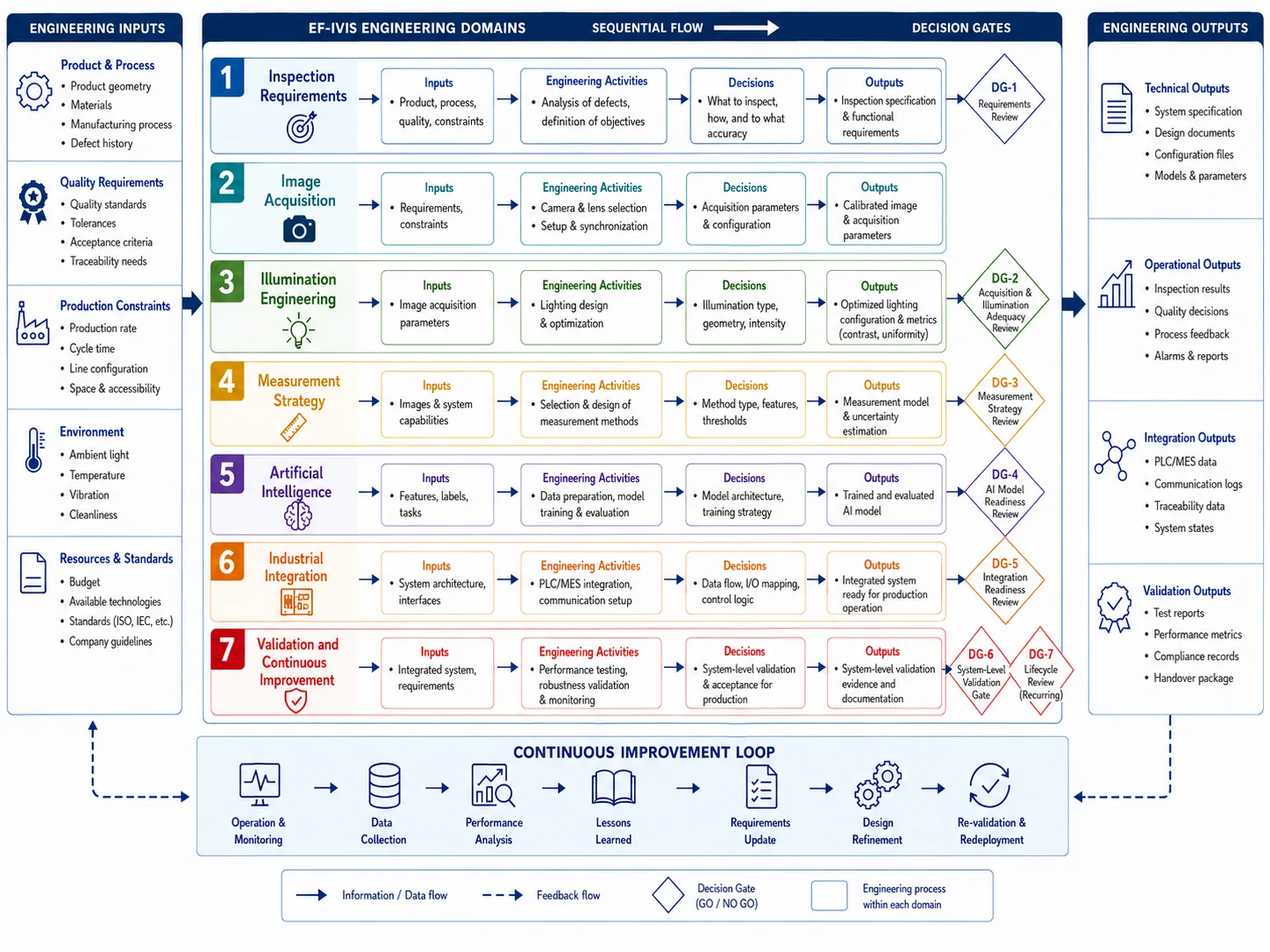
Systems engineering architecture of EF-IVIS.

Although presented sequentially, practical implementation remains iterative. Information generated within later engineering stages may require revision of previous design decisions, creating closed feedback loops supporting continuous engineering optimization [7,8,14,15,16,17,18,19]. The complete architecture of EF-IVIS is presented in Figure 4.

### 3.3. General Engineering Structure

Each engineering domain follows an identical methodological structure to ensure consistency throughout the framework [14,15,16,17,18,19]. Every engineering domain consists of six fundamental components:

- Engineering inputs;
- Engineering activities;
- Engineering decisions;
- Engineering outputs;
- Validation criteria;
- Engineering feedback.

**Figure 5:**
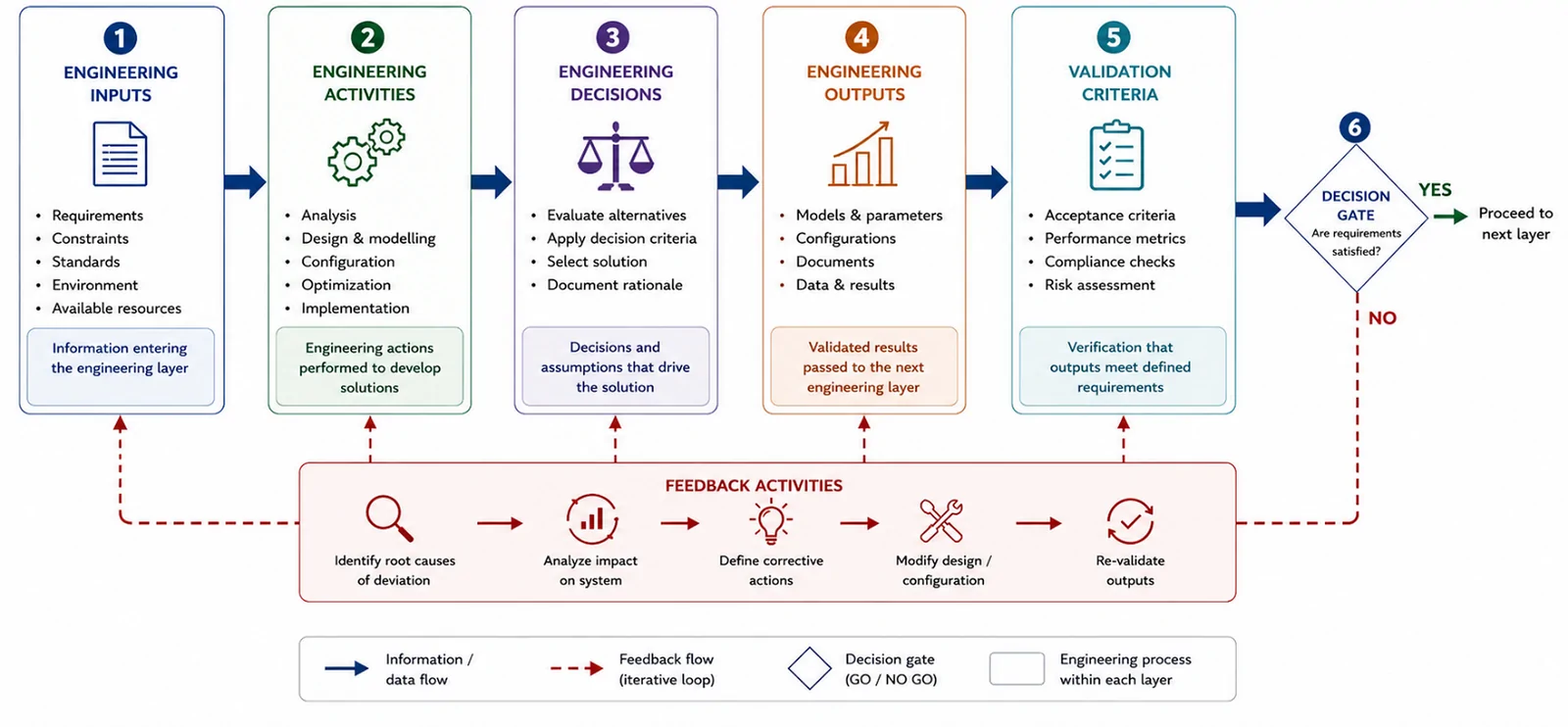
General engineering workflow adopted in EF-IVIS.

This common structure supports traceability of engineering decisions throughout the development process while facilitating iterative refinement whenever engineering objectives are not satisfied [14,15,16,17,18,19]. The general engineering workflow adopted within EF-IVIS is illustrated in Figure 5.

The relationship between individual methodological elements is summarized in Table 3.

### 3.4. Formal Representation of the Framework

To describe the nominal engineering logic underlying EF-IVIS, the output generated within engineering domain *i* may be represented conceptually as:

(1) $$O_{i}=\Phi (I_{i},A_{i},C_{i})$$

where:

$O_{i}$ denotes the engineering outputs;

$I_{i}$ represents engineering inputs;

$A_{i}$ denotes engineering activities;

$C_{i}$ represents engineering constraints and decision criteria.

In the nominal forward information flow, selected outputs from one domain may become inputs to one or more subsequent domains, while feedback may also return information to previously addressed domains. This relationship is a conceptual representation of information transfer and decision dependency rather than a mathematical prediction model and does not imply a strictly sequential process [14,15,16,17].

### 3.5. Engineering Domains Supporting EF-IVIS

The seven engineering domains constituting EF-IVIS originate from two complementary sources. First, they reflect the principal engineering activities consistently identified in the machine vision, industrial automation, and systems engineering literature. Their relationship with the experimental investigations is summarized in Table 4.

### 3.6. Framework Characteristics

Existing research provides mature engineering methods for many of the individual activities involved in intelligent vision inspection systems. Systems engineering establishes lifecycle-oriented processes for requirements definition, architecture development, implementation, verification, validation, deployment, and system evolution [14,15,16,17,18,19,53]. Requirements engineering provides structured methods for defining, analysing, documenting, and managing system requirements throughout the lifecycle [16]. Model-Based Systems Engineering and SysML support formal representation of requirements, system structure, behaviour, interfaces, and verification relationships [19]. These approaches constitute the general methodological foundation on which EF-IVIS is based.

Domain-specific methods are also well established. The machine vision literature addresses image formation, optical configuration, illumination, image processing, feature extraction, and inspection algorithms [26,27,28,29,30,31]. Artificial intelligence research provides methods for classification, defect detection, anomaly recognition, and segmentation [32,33,34,35,36,37,38,39,40,41,42]. Industrial metrology provides established principles for measurement strategy, traceability, uncertainty, and dimensional verification [47,48,49,50], whereas industrial automation and communication research addresses control integration, interoperability, synchronization, and production connectivity [1,2,3,4,5,6,7,8,9,10,11,20,21,22,23,24,25].

More specialized approaches have additionally been proposed for the development and integration of machine vision systems. Zhu et al. [54] proposed a reusable machine vision software framework for industrial product-appearance inspection, integrating image acquisition, configuration, database management, graphical interfaces, I/O communication, and execution architecture. The VDI/VDE/VDMA 2632 series [55,56] provides machine vision-specific guidance for requirement and system specifications and for acceptance assessment of classifying machine vision systems. More recently, Werheid et al. [57] proposed a conceptual framework for machine vision integration in manufacturing SMEs, structured around requirements and specifications, hardware setup, software setup, image analysis, and operational integration, including iterative loops and decision nodes.

These approaches demonstrate that neither multidisciplinary engineering nor the coordination of multiple technical activities is, by itself, novel. Accordingly, EF-IVIS does not claim novelty from the general idea of combining requirements, imaging, metrology, artificial intelligence, automation, and validation. The specific contribution of EF-IVIS lies instead in the way these established engineering activities are structured and connected for intelligent industrial vision inspection.

More specifically, EF-IVIS introduces four mutually related characteristics: (i) organization of the engineering lifecycle into seven explicitly defined IVIS domains; (ii) explicit representation of cross-domain dependencies through which decisions and outputs from one domain constrain activities in other domains; (iii) requirements-derived Decision Gates located between successive engineering domains; and (iv) an explicit feedback and domain-re-entry workflow when gate criteria are not satisfied. The separation of image acquisition and illumination engineering, the explicit incorporation of industrial metrology and AI within the same lifecycle, and the treatment of industrial integration as an engineering domain rather than a final implementation activity further specialize the framework for intelligent vision inspection.

EF-IVIS should therefore be interpreted as a domain-specific configuration and operationalization of established engineering principles, rather than as a new fundamental systems engineering theory or as the first multidisciplinary approach to machine vision development. Its specific methodological contribution concerns the proposed IVIS-specific domain structure, the defined cross-domain dependencies, the Decision Gate sequence, and the workflow connecting requirements, technical evidence, engineering decisions, corrective actions, and lifecycle feedback.

The relationship between EF-IVIS and the principal methodological reference approaches is summarized in Table 5. The purpose of the comparison is not to demonstrate general superiority of EF-IVIS but to identify differences in scope, engineering structure, dependency representation, and assessment logic.

The comparison indicates that several existing approaches already provide structured support for machine vision specification, implementation, integration, and acceptance. EF-IVIS therefore does not claim that such coordination is absent from the state of the art. Its distinctive methodological contribution is narrower: it defines an IVIS-specific seven-domain lifecycle in which cross-domain dependencies are explicitly identified and the progression between domains is controlled through requirements-derived Decision Gates. In this way, EF-IVIS complements rather than replaces general systems engineering, machine vision standards, and existing machine vision development frameworks. Its broader usability, repeatability, and effectiveness remain to be established prospectively.

The following section examines the empirical basis supporting the proposed EF-IVIS domain structure and cross-domain relationships. The analysis distinguishes direct experimental evidence obtained for selected technological domains from cross-cutting and methodological evidence supporting inspection requirements, system-level validation, and validation and continuous improvement. Because the same four investigations contributed to both the bottom-up development of EF-IVIS and its retrospective assessment, the resulting evidence should be interpreted as support for the relevance and internal consistency of the framework structure rather than as independent prospective validation of the complete methodology [43,44,45,46].

## 4. Experimental Support and Framework Evaluation

### 4.1. Framework Evaluation Philosophy

The evaluation of EF-IVIS differs from conventional experimental assessment of an individual machine vision algorithm or inspection device. Quantitative indicators such as classification accuracy, precision, recall, processing time, measurement uncertainty, and repeatability remain essential for evaluating specific technological solutions [26,27,28,29,30,31,32,33]. However, the present study addresses a domain-specific systems engineering framework whose structure also includes relationships between requirements, sensing, measurement, artificial intelligence, industrial integration, and lifecycle activities.

Accordingly, the evaluation adopted in this study considers two complementary levels of evidence. At the technological-domain level, results from the four industrial investigations provide experimental or industrial evidence concerning selected EF-IVIS domains [43,44,45,46]. At the framework level, the analysis examines whether recurring engineering observations and dependencies identified across the investigations are consistent with the proposed domain structure and with established systems engineering principles [14,15,16,17,18,19].

The four investigations should not be interpreted as independent validation experiments designed after EF-IVIS had been formulated. They contributed to the bottom-up development of the framework and are therefore used here for retrospective evidence mapping. Their role is to provide empirical grounding for selected technological domains and to support assessment of the internal consistency and practical relevance of the overall structure.

Independent prospective validation of EF-IVIS would require application of the framework from the initial requirements stage throughout the complete lifecycle of a new industrial inspection project, preferably with predefined assessment criteria and independent or comparative evaluation. Such validation remains a subject of future work.

**Figure 6:**
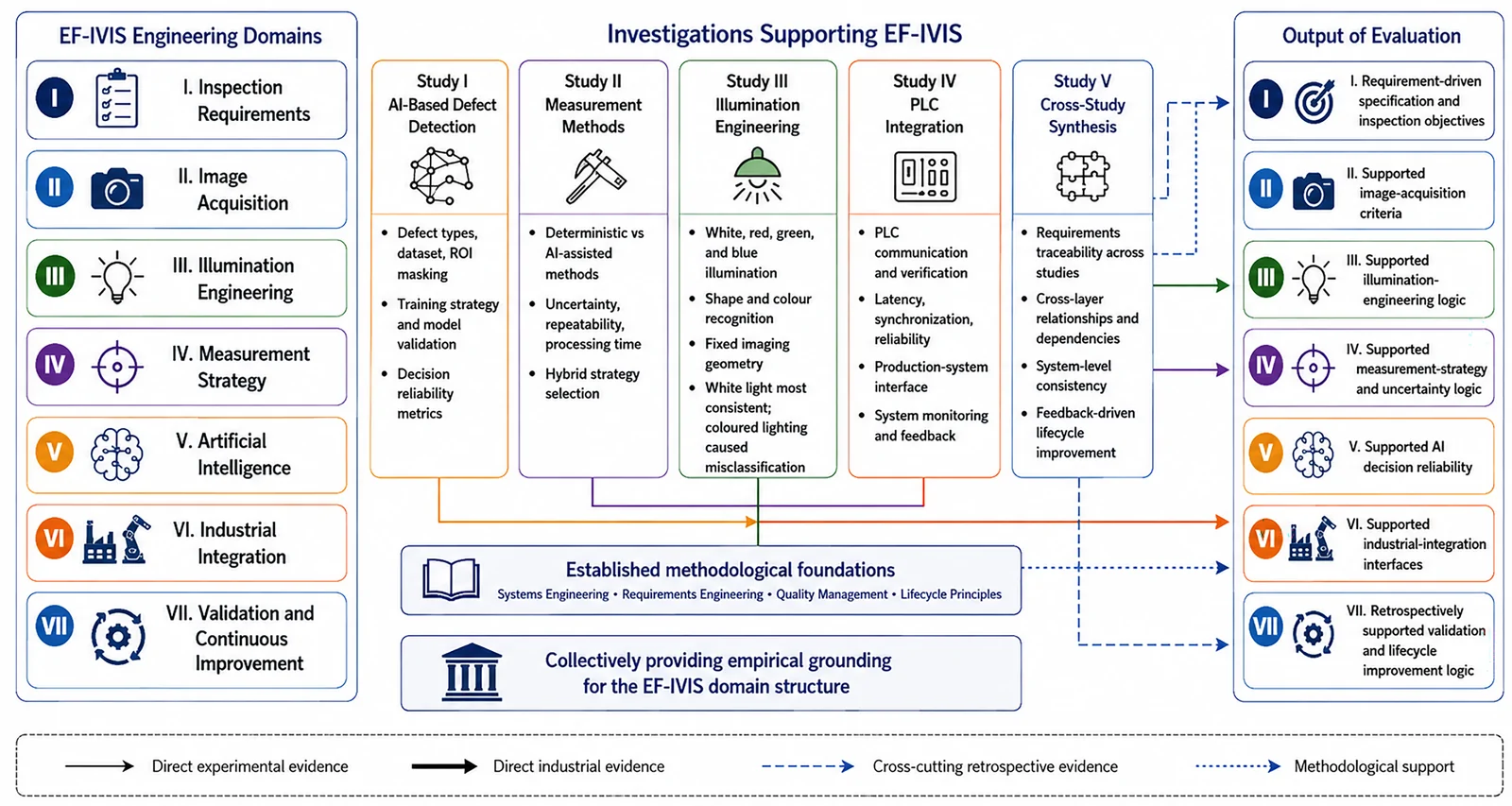
Evidence integration and retrospective framework evaluation strategy adopted for EF-IVIS, based on the industrial investigations [43,44,45,46] and established methodological foundations [12,13,14,15,16,17,18,19].

The evidence integration and framework evaluation strategy adopted in this study is summarized in Figure 6.

Figure 6 summarizes the evidence integration and framework evaluation strategy adopted for EF-IVIS. Evidence derived from the four industrial investigations [43,44,45,46] is complemented by cross-study synthesis and established methodological foundations [12,13,14,15,16,17,18,19]. The mapping distinguishes direct experimental evidence, direct industrial evidence, cross-cutting retrospective evidence, and methodological support. Direct evidence is assigned to selected technological domains, whereas inspection requirements and validation and continuous improvement are supported primarily through cross-study observations and established engineering principles. This differentiated evidence structure enables assessment of the empirical grounding, internal consistency, and cross-domain relationships of EF-IVIS without implying independent prospective validation of the complete framework.

The detailed criteria used to assign these forms of evidence to individual EF-IVIS domains are described in Section 4.2.

### 4.2. Evidence Mapping Methodology

The retrospective evidence-mapping process was used to assess the extent to which the proposed EF-IVIS engineering domains and cross-domain relationships are supported by the four industrial investigations [43,44,45,46]. The purpose of this analysis was not to provide independent prospective validation of the complete EF-IVIS lifecycle. Instead, the mapping was intended to determine whether the engineering observations obtained during the coordinated research programme provide direct or cross-cutting support for the proposed domain structure and whether these observations remain consistent with established systems engineering principles [14,15,16,17,18,19].

Because the same four investigations contributed to the bottom-up development of EF-IVIS and to its subsequent retrospective assessment, the evidence was classified according to its relationship with the individual framework domains. Four complementary types of support were distinguished:

1. Direct experimental evidence was assigned when an investigation explicitly examined engineering variables, performance indicators, or implementation constraints corresponding to a specific EF-IVIS technological domain and reported experimental or industrial observations concerning their influence on inspection performance.
2. Direct industrial evidence was assigned when an investigation evaluated implementation, communication, control, synchronization, or system behaviour within an integrated industrial or production-control architecture.
3. Cross-cutting retrospective evidence was assigned when a framework domain or dependency was supported by recurring observations across multiple investigations but was not itself the subject of a dedicated domain-specific experiment.
4. Methodological support was assigned when a framework element was primarily derived from established systems engineering, requirements engineering, quality management, or lifecycle principles and was consistent with the engineering practices observed across the industrial investigations [12,13,14,15,16,17,18,19].

The mapping procedure considered, for each investigation, the original engineering objective, the technological variables investigated, the measured or observed outputs, the industrial constraints considered, and the conclusions supported by the reported results. An investigation was mapped to a domain as direct experimental or direct industrial evidence only when the corresponding engineering activity was explicitly examined or implemented within the original study. Broader implications inferred through comparison of several investigations were classified as cross-cutting evidence rather than direct experimental support. This distinction was introduced to avoid attributing stronger empirical support to EF-IVIS domains than is justified by the underlying studies.

The first investigation addressed artificial intelligence-based defect detection in an industrial machine vision application [43]. It examined engineering aspects associated with image acquisition, region-of-interest definition and masking, dataset preparation, model training, defect classification, and industrial applicability. The study therefore provides direct experimental evidence for the artificial intelligence domain and additional supporting observations concerning the dependence of AI performance on image quality and data preparation.

The second investigation compared deterministic measurement functions with artificial intelligence-assisted approaches implemented within an industrial vision system [44]. The experimental results demonstrated differences in measurement functionality, adaptability, processing time, and suitability for different inspection tasks. The study also indicated that deterministic and AI-assisted approaches can provide complementary capabilities, supporting the treatment of measurement strategy as a distinct EF-IVIS engineering domain and motivating the consideration of hybrid inspection strategies. These findings are consistent with established industrial-metrology principles [47,48,49,50].

The third investigation examined the influence of illumination conditions on vision-based inspection performance [45]. The experiments compared white, red, green, and blue illumination during shape and colour recognition. White illumination produced the most consistent and unbiased detection, whereas coloured illumination resulted in application-dependent changes in feature visibility and, in several cases, incorrect colour or object classification. These observations provide direct experimental evidence that illumination selection can influence the reliability of acquired visual information and subsequent inspection results, thereby supporting the image acquisition and illumination engineering domains of EF-IVIS.

The fourth investigation addressed industrial integration by connecting machine vision inspection with programmable logic control and industrial communication infrastructure [46]. The study evaluated inspection performance together with PLC-supported verification, communication timing, synchronization, and production-system interaction. The results showed that industrial suitability depends not only on image-processing performance but also on communication reliability, deterministic execution, and coordination with manufacturing control systems. The investigation therefore provides direct industrial evidence for the industrial integration domain and is consistent with current developments in Industry 4.0 and cyber-physical production systems [1,2,3,4,5,6,9,10,11,20,21,22,23,24,25].

Inspection requirements were not examined through a separate dedicated experiment. Their inclusion in EF-IVIS is supported retrospectively by the observation that all four investigations originated from defined industrial inspection objectives, product characteristics, acceptance requirements, and implementation constraints [43,44,45,46], together with established requirements engineering and systems engineering principles [12,13,14,15,16,17,18,19]. This support is therefore classified as cross-cutting and methodological rather than direct experimental evidence.

Similarly, validation and continuous improvement were not evaluated prospectively throughout the complete lifecycle of a newly developed inspection system. Evidence for this domain is derived from recurring verification, adjustment, performance assessment, and feedback activities reported across the four investigations, together with established systems engineering and quality management principles [12,13,14,15,16,17,18,19,43,44,45,46]. The resulting support should therefore be interpreted as integrated and cross-domain evidence rather than as independent experimental validation of the complete lifecycle process.

The resulting evidence mapping is summarized in Table 6.

Collectively, the four investigations provide direct experimental or industrial evidence for selected technological domains of EF-IVIS, including illumination engineering, measurement strategy, artificial intelligence, and industrial integration, together with supporting experimental observations relevant to image acquisition. Inspection requirements and validation and continuous improvement are supported primarily through cross-study synthesis and established engineering principles. The evidence mapping therefore provides a structured assessment of the empirical grounding, internal consistency, and practical relevance of the proposed framework structure, but it does not constitute independent prospective validation of EF-IVIS as a complete systems engineering methodology.

To make the empirical basis of this assessment explicit, Table 7 summarizes the experimental setup, dataset or test scope, reported performance indicators, principal results, available information on variability or uncertainty, and the specific EF-IVIS proposition supported by each investigation. Because the four studies differ substantially in experimental design, objectives, and reporting depth, the original forms of evidence are retained rather than converted into a common performance metric [43,44,45,46].

As shown in Table 7, the empirical basis supporting EF-IVIS is heterogeneous. Investigations [43,44,46] provide quantitative evidence for selected technological and integration decisions, whereas investigation [45] provides controlled comparative evidence concerning the effect of illumination conditions without reporting a statistical performance dataset. The available studies also differ in sample size, reported performance indicators, and treatment of variability and uncertainty. These differences are retained explicitly in the framework evaluation to avoid treating the four investigations as methodologically equivalent or assigning a stronger level of evidence than is supported by the original studies.

The following section examines how this differentiated evidence supports the individual EF-IVIS engineering domains and the cross-domain relationships between them.

### 4.3. Evidence Supporting the Engineering Domains

#### 4.3.1. Cross-Cutting Evidence for Inspection Requirements

The first engineering domain of EF-IVIS concerns the specification of inspection requirements, which determine the overall architecture and functionality of the inspection system. Systems engineering standards consistently emphasize that engineering decisions should originate from clearly defined functional and performance requirements rather than from available technological solutions [14,15,16,17,18,19,43,44,45,46]. Similar principles are adopted in industrial quality management, where inspection planning begins with the identification of critical quality characteristics, dimensional tolerances, acceptance criteria, and production constraints [12,13].

Within the coordinated experimental programme, all four investigations originated from industrial inspection problems defined before selecting image acquisition hardware, measurement algorithms, or artificial intelligence models. Although each study addressed different technological challenges, the engineering workflow consistently followed the same sequence: identification of inspection objectives, analysis of product characteristics, definition of measurable quality parameters, and selection of appropriate inspection methodologies [43,44,45,46].

This observation supports one of the fundamental assumptions of EF-IVIS, namely that engineering requirements represent the primary driver of system architecture. Rather than selecting technologies first and adapting inspection objectives afterwards, the experimental studies demonstrated that inspection requirements constrain all subsequent engineering decisions, including optical configuration, image acquisition strategy, artificial intelligence implementation, communication architecture, and validation procedures.

From an engineering perspective, this finding is consistent with established systems engineering methodologies [14,15,16,17,18,19,43,44,45,46], which recommend maintaining full traceability between stakeholder requirements, system functions, design solutions, verification activities, and operational validation. Accordingly, the proposed framework introduces the inspection requirements domain as the entry point for all engineering activities.

The engineering activities associated with this domain include:

- Definition of inspection objectives;
- Identification of critical product characteristics;
- Specification of dimensional and functional tolerances;
- Determination of environmental constraints;
- Definition of production performance requirements;
- Establishment of validation criteria.

These activities generate a structured engineering specification that becomes the input for all subsequent EF-IVIS domains. Inspection requirements therefore act as the entry domain of the framework, defining the functional, performance, environmental, and acceptance constraints that guide subsequent decisions concerning image acquisition, illumination, measurement strategy, artificial intelligence, industrial integration, and system-level validation.

To prevent downstream engineering decisions from being based on incomplete or insufficiently defined requirements, EF-IVIS introduces Decision Gate DG-1. This gate requires the completeness and consistency of inspection requirements to be assessed before subsequent image acquisition and system design activities proceed. The specific acceptance criteria remain application-dependent and should reflect the defined inspection objectives, product characteristics, production constraints, and required validation conditions.

#### 4.3.2. Experimental Support for Image Acquisition and Illumination

Image acquisition and illumination constitute closely related engineering domains because the reliability of subsequent inspection depends on the quality and stability of the visual information acquired by the system. The established machine vision literature emphasizes the importance of sensor characteristics, optical configuration, illumination, calibration, and image quality for reliable inspection [26,27,28,29,30,31]. Similarly, the performance of data-driven inspection methods depends on the consistency and representativeness of the acquired image data [36,37,38,39,40,41,42].

Direct experimental evidence concerning illumination was obtained in investigation [45]. The study evaluated shape and colour recognition under four illumination conditions: white, red, green, and blue light. To reduce the influence of geometric variability, the object positions and focal distance were kept fixed during the experiments, while the camera exposure was initially set to 5 ms and the gain to 1.0×. White balance was additionally configured for colour reproduction under white illumination [45].

The results demonstrated a clear dependence of inspection performance on illumination conditions. Under white light, the detected object positions and colours corresponded correctly to the actual objects. Under red illumination, object shapes were detected correctly, but the green object was misclassified as red. Under green illumination, only green and, in some cases, yellow objects were correctly identified, while the shape of the orange object was not always detected correctly. Under blue illumination, all detected objects were classified as green, and the orange object was not detected [45]. These findings demonstrate that illumination characteristics can directly affect feature visibility and classification reliability in vision inspection systems.

The investigation therefore provides direct experimental support for treating illumination engineering as a distinct EF-IVIS domain. It also provides supporting evidence for the image acquisition domain because illumination conditions influence the visual information available to subsequent processing stages. However, the study did not independently evaluate the effects of camera type, lens selection, spatial resolution, or calibration accuracy. Support for these aspects of image acquisition is therefore derived primarily from the established machine vision literature [26,27,28,29,30,31] rather than from investigation [45] alone.

Within EF-IVIS, image acquisition and illumination engineering are treated as separate but strongly interacting domains. Image acquisition defines the sensing and optical configuration required to obtain usable image information, whereas illumination engineering concerns the controlled generation of optical conditions that make relevant product features sufficiently distinguishable for subsequent measurement or classification. Their separation allows illumination-related limitations to be identified and corrected before downstream measurement or artificial intelligence methods are selected.

To support engineering decision-making, the framework considers image quality in terms of application-specific indicators such as feature visibility, contrast, signal-to-noise ratio, spatial resolution, repeatability, and acquisition stability [26,27,28,29,30,31]. These indicators form the basis of Decision Gate DG-2, which assesses whether the acquired visual information is sufficiently stable and informative for subsequent measurement or decision-making. The numerical acceptance thresholds are not universal and must be defined according to the specific inspection requirements and production conditions. The principal engineering activities associated with image acquisition and illumination, together with their downstream influence and supporting evidence, are summarized in Table 8.

The available evidence therefore supports the treatment of image acquisition as an engineering domain whose configuration influences the information available to subsequent inspection activities. Investigation [45] provides direct experimental evidence for the effect of illumination conditions and supporting evidence for selected acquisition-related dependencies, while broader aspects of image acquisition remain supported primarily by the established machine vision literature [26,27,28,29,30,31].

#### 4.3.3. Experimental Support for Measurement Strategy

The measurement strategy domain defines the engineering methodology through which image information is transformed into quantitative quality indicators supporting industrial decision-making. Unlike traditional machine vision approaches, where measurement is frequently interpreted as the final stage of image processing, EF-IVIS considers measurement strategy as an independent engineering domain requiring systematic selection of measurement principles, uncertainty analysis, validation procedures, and decision criteria.

Contemporary industrial metrology emphasizes that measurement performance depends not only on the applied algorithm but also on the complete measurement chain, including image acquisition, calibration, environmental conditions, uncertainty estimation, and traceability [47,48,49,50]. These principles are reflected in the ISO GPS framework and the Guide to the Expression of Uncertainty in Measurement (GUM), which recommend considering the entire measurement process rather than isolated numerical results [48,49,50].

Investigation [44], performed using the Cognex In-Sight D900 vision system, provides direct experimental evidence supporting the treatment of measurement strategy as a distinct engineering domain. The study compared deterministic user-configured inspection functions with AI-assisted ViDi tools and demonstrated differences in the type of inspection information generated, configuration requirements, processing time, and sensitivity of the resulting outputs to changes in object displacement and rotation. The results indicate that deterministic and AI-assisted approaches provide different engineering capabilities and may be considered complementary depending on the inspection objective and production constraints [44].

Within EF-IVIS, selection of a measurement or inspection strategy should be based on application-specific engineering criteria. These may include the type of information required from the inspection, feature and geometry characteristics, processing-time constraints, required measurement uncertainty where quantitative metrology is involved, and robustness under the expected operating conditions [44,47,48,49,50]. Investigation [44] provides direct evidence for selected aspects of this decision, particularly method functionality, processing time, configuration characteristics, and sensitivity to object displacement and rotation, whereas uncertainty and traceability requirements are derived primarily from established industrial-metrology principles [47,48,49,50].

Investigation [44] supports consideration of hybrid inspection strategies in applications where deterministic functions and AI-assisted methods provide complementary information. Such an approach may combine explicitly configured measurement or feature-based results with broader deviation detection, although the performance of a complete hybrid configuration was not independently quantified in the reported investigation.

This interpretation is consistent with recent developments in industrial metrology, where optical measurement systems increasingly integrate data-driven analytical methods while preserving traceability and measurement reliability [47,50]. Within EF-IVIS, the measurement strategy domain therefore performs three principal engineering functions:

- Transformation of image information into engineering measurements;
- Selection of inspection methodology;
- Estimation of measurement confidence.

Before proceeding to intelligent decision-making, the generated measurement data are evaluated using Decision Gate DG-3, which verifies compliance with predefined uncertainty limits, repeatability criteria, and production requirements. These criteria define the intended scope of DG-3 and are application-specific. Investigation [44] provides direct experimental evidence for selected method-selection and stability considerations, but it does not constitute a comprehensive experimental validation of measurement uncertainty, traceability, or all DG-3 acceptance criteria.

The available evidence supports treating measurement strategy as a distinct EF-IVIS engineering domain rather than solely as a computational stage of image processing. Investigation [44] demonstrates that deterministic and AI-assisted approaches differ in the type of information generated, configuration requirements, computational demand, and sensitivity to inspection conditions. These differences make strategy selection an engineering decision that should be based on the inspection objective and production constraints rather than on an assumed universal hierarchy of methods. The principal evidence-based trade-offs are summarized in Table 9.

#### 4.3.4. Direct Experimental Evidence for the Artificial Intelligence Domain

Artificial intelligence constitutes an important technological component of modern industrial vision inspection systems. Within EF-IVIS, however, artificial intelligence is treated as one engineering domain within a broader system rather than as the primary determinant of inspection performance. This systems-oriented perspective reflects the fact that successful industrial deployment of AI-based inspection depends not only on the selected model or learning algorithm but also on the quality and consistency of acquired image data, preprocessing, dataset preparation, computational resources, and compatibility with the surrounding industrial system [32,33,34,35,36,37,38,39,40,41,42].

Recent developments in deep learning, explainable artificial intelligence, and Vision Transformer architectures further demonstrate the increasing capabilities of data-driven inspection methods [37,38,39,40,41,42,58]. At the same time, their industrial application introduces additional engineering requirements associated with training-data preparation, model configuration, performance verification, computational demand, repeatability, and deployment within production environments. EF-IVIS therefore treats the selection and implementation of artificial intelligence as an engineering decision that must remain consistent with upstream acquisition and measurement decisions and with downstream production constraints.

Investigation [43] provides direct experimental evidence for this domain. The study used a Cognex In-Sight D900 smart camera and the ViDiDetect Red Analyze tool in unsupervised mode to inspect the machined surface of an automotive suspension knuckle for defects associated with burrs, chips, and cutting-tool wear. The experimental workflow included image acquisition, definition of the region of interest, preparation of labelled image data, model training, defect scoring, and Pass/Fail classification [43].

The dataset used for training and testing consisted of 500 labelled images. Approximately 80% of the images were assigned to training, with the remaining data divided between validation and testing. The ViDiDetect implementation returned a deviation score associated with the detected defect area and a Boolean Pass/Fail result [43].

An important observation from investigation [43] concerned the definition of the region of interest. A manually defined mask was introduced to restrict the analysed region to the relevant edge of the machined surface and to exclude image areas that were not relevant to the intended inspection objective. The reported results showed that this modification improved the classification outcome. In the post-mask assessment, classification limits of 6.32 mm^2^ for good classification and 6.65 mm^2^ for bad classification were reported [43].

These findings provide direct experimental support for the EF-IVIS principle that AI-based inspection should not be considered independently from the engineering preparation of input data. In particular, investigation [43] demonstrates that ROI definition, masking, and preparation of image data can materially influence the output of a data-driven inspection method. The evidence therefore supports the artificial intelligence domain while simultaneously illustrating its dependency on decisions made during preceding acquisition and data-preparation activities.

Within EF-IVIS, the principal engineering activities associated with the artificial intelligence domain include:

- Preparation and verification of representative image datasets;
- Definition of regions of interest and relevant preprocessing procedures;
- Selection of an appropriate AI methodology;
- Model training and configuration;
- Evaluation of model outputs against predefined inspection requirements;
- Assessment of computational and deployment constraints;
- Integration of the resulting decision logic with the industrial inspection system.

Artificial intelligence is not required for every inspection problem. Deterministic image-processing and measurement functions remain appropriate where inspected characteristics can be explicitly defined and evaluated through stable geometric, dimensional, or feature-based criteria. AI-assisted methods become particularly relevant when the inspection objective involves visual variability, surface anomalies, complex defect patterns, or features that are difficult to represent through fixed deterministic rules. Investigation [44] additionally indicates that deterministic and AI-assisted methods can provide complementary capabilities rather than representing mutually exclusive alternatives.

EF-IVIS therefore supports the use of hybrid inspection strategies when the inspection objective benefits from combining explicitly configured measurement or feature-based functions with data-driven anomaly detection. The selection between deterministic, AI-assisted, and hybrid approaches should be based on inspection requirements, characteristics of the available data, interpretability needs, computational constraints, required processing time, and expected production conditions rather than on the availability of a particular AI technology.

Decision Gate DG-4 provides the assessment point between AI model development and subsequent industrial integration. The gate evaluates whether the selected AI-based inspection solution provides sufficient evidence of suitability for the intended application. Depending on the inspection task, the assessment may include classification performance, robustness to relevant input variability, repeatability, processing time, computational requirements, explainability or interpretability, and compatibility with production constraints.

The numerical acceptance limits associated with DG-4 are application-specific and should be derived from the inspection requirements established earlier in the EF-IVIS process. Investigation [43] provides direct evidence concerning dataset preparation, ROI definition, masking, defect scoring, and Pass/Fail classification, but it does not constitute a comprehensive experimental evaluation of all DG-4 criteria. Broader robustness across independent datasets, long-term model drift, cross-platform performance, and lifecycle behaviour were not evaluated in the reported investigation and therefore require application-specific verification during prospective use of EF-IVIS.

Overall, the evidence from investigation [43] supports the treatment of artificial intelligence as a distinct but interdependent EF-IVIS engineering domain. The investigation demonstrates that the outcome of AI-based inspection depends not only on the selected learning method but also on the engineering preparation of input data and the definition of the analysed region. More broadly, the artificial intelligence domain must remain consistent with inspection requirements, upstream acquisition and measurement decisions, computational constraints, and downstream industrial integration. AI-based inspection is therefore treated within EF-IVIS as one component of a coordinated engineering process rather than as an independently optimized technological solution.

#### 4.3.5. Direct Industrial Evidence for the Industrial Integration Domain

Industrial deployment represents the stage at which the vision inspection system becomes operationally connected with the manufacturing environment. Within EF-IVIS, industrial integration is therefore treated as a distinct engineering domain rather than as a final implementation activity performed after image-processing development has been completed. Modern industrial inspection systems interact with programmable logic controllers, production equipment, industrial communication networks, supervisory systems, and manufacturing information systems, making communication and synchronization part of the overall inspection function [1,2,3,4,5,6,20,21,22,23,24,25].

This perspective is consistent with Industry 4.0 and cyber-physical production-system concepts, in which sensing, decision-making, communication, and control form interconnected elements of the manufacturing system [1,2,3,4,5,6,20,21,22,23,24,25]. Standards and industrial architectures such as IEC 61131, IEC 62264, and OPC UA provide established mechanisms for automation-system integration and information exchange [20,21,22,23,24,25]. EF-IVIS does not replace these technologies or standards; instead, it incorporates their application into the engineering lifecycle of the vision inspection system.

Investigation [46] provides direct industrial evidence for the industrial integration domain. The study integrated a Cognex In-Sight 2802C smart camera with an Allen-Bradley Compact GuardLogix PLC through EtherNet/IP implicit cyclic communication. Three different inspection cases were evaluated, involving 3D-printed holders, automotive electrical connectors, and fibre-optic tubes [46].

The investigation demonstrated that industrial inspection performance depends on more than camera-level classification capability. The integrated architecture required coordinated image acquisition, inspection execution, communication with the PLC, controller-level verification, and transfer of inspection decisions to the surrounding manufacturing process. The study therefore provides empirical evidence that communication architecture and controller logic can influence the reliability and timing of the complete inspection system.

Quantitative results reported in [46] provide a direct basis for this interpretation. Detection accuracy exceeded 95% across the investigated cases, while PLC-level triple verification reduced the false-positive rate from 7.5% to 5.4%, corresponding to a 28% relative reduction compared with camera-only classification. Mean end-to-end response times of 54.7 ± 6.3 ms and 58.9 ± 8.1 ms were reported for two investigated task categories. The system also achieved a reported throughput of approximately 700–950 parts/min for short-cycle operations [46].

The investigation additionally examined system behaviour under increased network load and selected environmental perturbations. At 50% network-bandwidth saturation, latency increased by less than 6%. Under ±20% changes in ambient illumination and minor dust deposition on the lens cover, classification accuracy remained above 93%, although the false-positive rate increased by approximately 2–3% [46]. These results provide evidence that integration assessment should consider not only nominal communication functionality but also timing variability and robustness under relevant operating disturbances.

Within EF-IVIS, the industrial integration domain therefore encompasses the following engineering activities:

- Definition and implementation of industrial communication interfaces;
- Synchronization of image acquisition, inspection, and production events;
- Integration with programmable logic controllers and production equipment;
- Transfer and management of inspection results;
- Verification of communication timing and data consistency;
- Implementation of production decision and feedback logic;
- Assessment of integration robustness under expected operating conditions.

The outputs of this domain include an implemented and verified communication architecture, synchronized inspection and production workflows, defined decision interfaces, and evidence demonstrating that the integrated system can satisfy application-specific timing, reliability, and production requirements.

**Figure 7:**
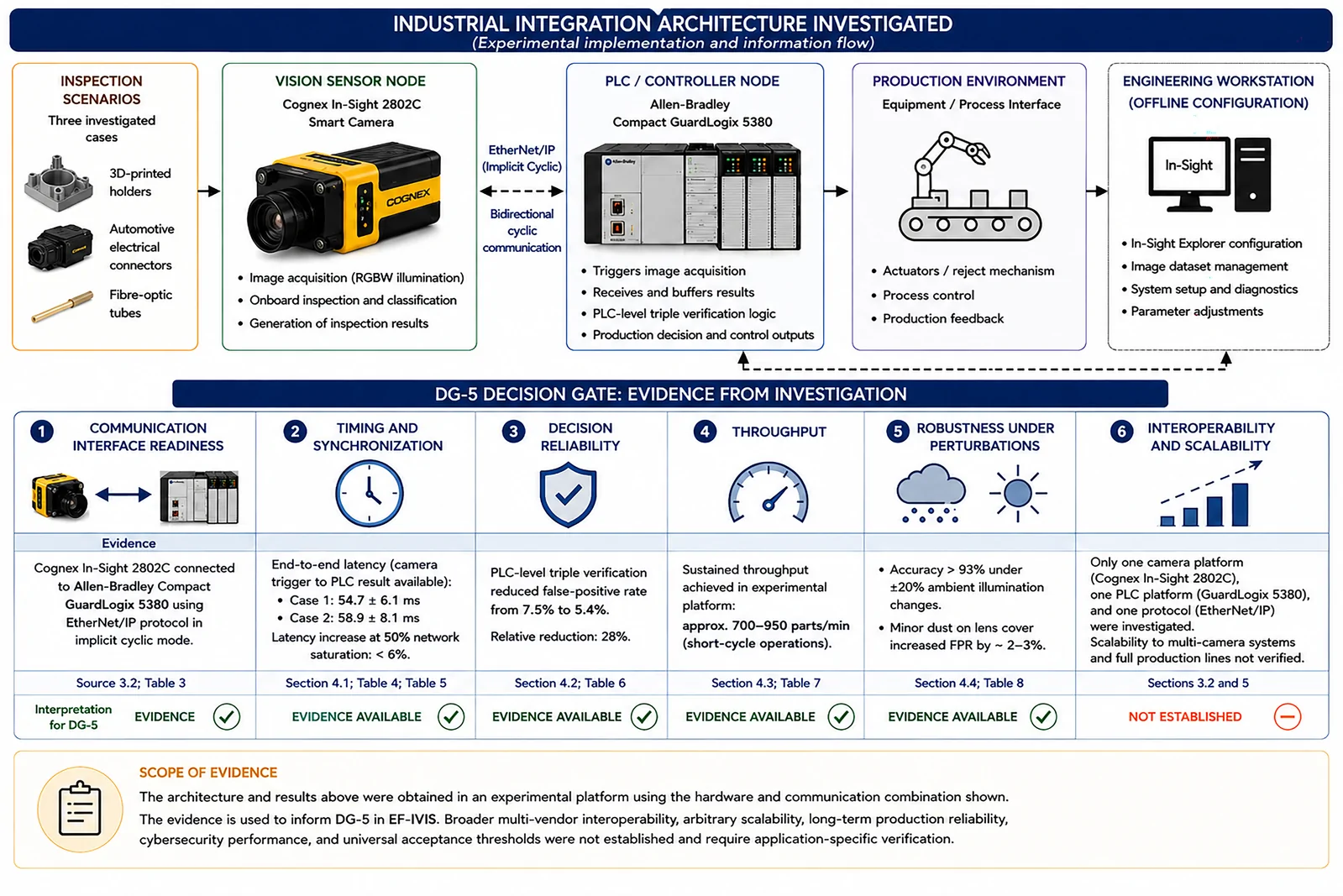
Experimentally implemented industrial integration architecture and DG-5 assessment based on investigation [46].

The experimentally implemented integration architecture reported in investigation [46], together with the principal categories of evidence relevant to the subsequent DG-5 assessment, is summarized in Figure 7.

The results of investigation [46] provide direct industrial evidence supporting the treatment of industrial integration as a distinct EF-IVIS engineering domain. In particular, the study provides implementation and quantitative evidence concerning communication-interface readiness, timing and synchronization, controller-level decision verification, production throughput, and system behaviour under selected operating perturbations. At the same time, the investigated configuration does not establish broad multi-vendor interoperability or scalability beyond the evaluated camera, controller, and communication architecture.

EF-IVIS introduces Decision Gate DG-5 to assess whether the integrated inspection architecture provides sufficient evidence to proceed toward system-level validation and production deployment. DG-5 considers communication-interface readiness, timing and synchronization, decision reliability, production throughput, robustness under relevant operating perturbations, and interoperability and scalability within the intended manufacturing environment.

To demonstrate the complete operational logic of a Decision Gate, DG-5 was applied retrospectively to the industrial evidence reported in investigation [46]. Because investigation [46] preceded the formalization of EF-IVIS and was not originally designed as a prospective Decision Gate experiment, numerical acceptance thresholds were not predefined in that study. For the present worked example, an application-specific acceptance specification is therefore defined before the reported evidence is evaluated against the gate. These limits are used solely to demonstrate the complete and reproducible DG-5 procedure and should not be interpreted either as universal EF-IVIS thresholds or as requirements originally reported in investigation [46].

For this worked application, the mandatory integration requirements are defined as follows: mean end-to-end response time ≤ 70 ms, latency increase under 50% network-bandwidth saturation ≤ 10%, false-positive rate ≤ 6.0%, production throughput ≥ 700 parts/min, and classification accuracy ≥ 93% under the investigated environmental perturbations. Successful communication through the intended Cognex–Allen-Bradley EtherNet/IP architecture is additionally treated as a mandatory functional requirement. Broader multi-vendor interoperability is outside the deployment scope of this worked application and is therefore not treated as a mandatory DG-5 criterion. The complete application of DG-5, including predefined acceptance requirements, measured evidence, assessment outcomes, corrective action, and re-evaluation, is summarized in Table 10.

The worked example demonstrates the complete sequence from a predefined requirement to measurement, assessment, corrective action, and re-evaluation. For each mandatory DG-5 criterion, the measured or verified result is compared directly with the corresponding application-specific acceptance requirement. A criterion is classified as PASS only when the predefined requirement is satisfied. If any mandatory criterion is classified as FAIL, progression to the subsequent engineering stage is not permitted, and corrective action must be implemented within the affected domain. The failed criterion is then re-evaluated against the same acceptance requirement. A CONDITIONAL outcome may be applied only to explicitly identified non-critical deviations for which corrective action and subsequent verification are documented and where the deviation does not compromise any mandatory system requirement.

In the worked DG-5 case, the initial camera-only false-positive rate of 7.5% exceeded the application-specific acceptance limit of 6.0%, resulting in a FAIL decision for the decision reliability criterion. PLC-level triple verification was therefore introduced as a corrective action. Subsequent re-evaluation showed that the false-positive rate decreased to 5.4%, thereby satisfying the predefined requirement and changing the criterion outcome to PASS. Because the remaining mandatory DG-5 criteria also satisfied their respective acceptance requirements, the final Decision Gate outcome was PASS, permitting progression to system-level validation.

The numerical limits used in this worked example are application-specific and are not proposed as universal EF-IVIS thresholds. In prospective EF-IVIS applications, equivalent acceptance limits should be established in the inspection requirements domain before the corresponding technical solution is evaluated. The purpose of the Decision Gates is therefore not to prescribe universal numerical values, but to provide a reproducible engineering process linking predefined requirements, technical evidence, Pass/Fail assessment, corrective action, and re-verification.

#### 4.3.6. Cross-Cutting Evidence for Continuous Improvement

One of the distinguishing characteristics of EF-IVIS is the explicit incorporation of continuous engineering improvement as an integral framework component. Rather than treating validation as a one-time activity, the framework assumes that engineering knowledge may continue to evolve during implementation and industrial operation.

This perspective is consistent with systems engineering principles, quality management methodologies, and Smart Manufacturing concepts, which emphasize iterative improvement based on operational feedback [1,2,3,4,5,6,9,10,11,12,13,14,15,16,17,18,19,51].

Analysis of the coordinated research programme indicated that the investigations generated observations extending beyond their immediate domain-specific objectives. Illumination studies informed measurement considerations; measurement investigations identified constraints relevant to artificial intelligence implementation; AI-based studies highlighted dependencies related to dataset preparation and image-region definition; and industrial integration introduced additional considerations associated with communication reliability, synchronization, and production-system interaction [43,44,45,46].

These observations indicate that engineering knowledge generated within one domain may influence decisions made in other domains. EF-IVIS therefore incorporates a structured feedback logic through which operational observations can initiate analysis, identification of the affected engineering domain, corrective action, and subsequent revalidation.

The validation and continuous improvement domain includes:

- Operational performance monitoring;
- Analysis of inspection failures;
- Identification of process variability;
- Update of engineering requirements;
- Optimization of inspection parameters;
- Retraining of artificial intelligence models where required;
- Refinement of validation criteria.

Continuous improvement may therefore involve changes to optical configurations, measurement methodologies, AI models, communication architectures, or engineering requirements while maintaining consistency across the overall framework.

**Figure 8:**
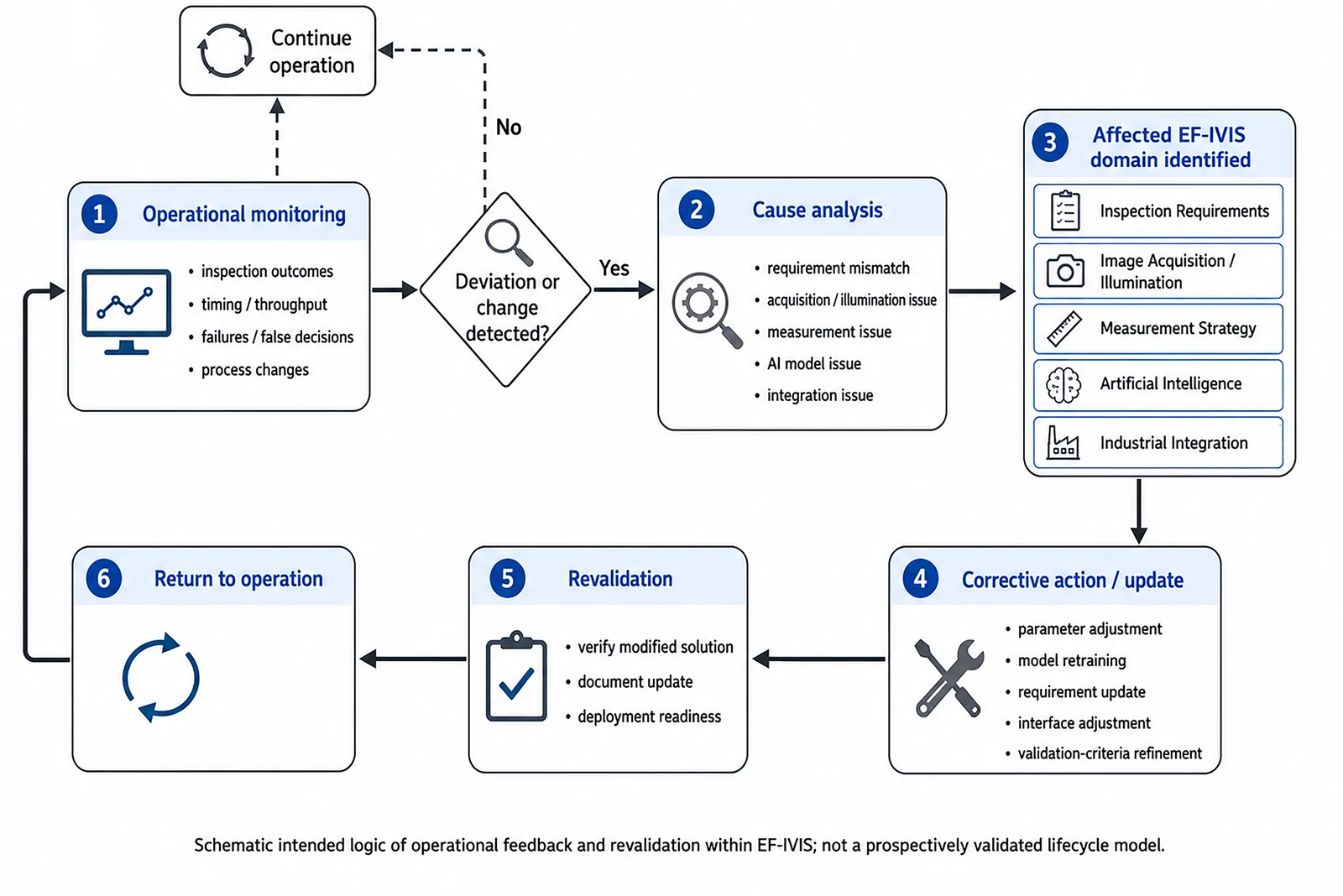
Simplified feedback logic linking operational monitoring, cause analysis, corrective action, and revalidation within EF-IVIS.

The simplified feedback logic is illustrated in Figure 8. Operational monitoring provides the starting point for detecting deviations or changes. When corrective action is required, the affected EF-IVIS domain is identified, the necessary modification is implemented, and the modified solution is revalidated before returning to operation.

Cross-study analysis suggests that such structured feedback may support adaptation to changing production conditions, product modifications, and evolving quality requirements. However, the present study does not provide prospective lifecycle evidence demonstrating the long-term effectiveness of this feedback mechanism. Its quantitative benefits, repeatability, and long-term effectiveness therefore require future longitudinal validation.

### 4.4. Cross-Domain Engineering Dependencies

The coordinated research programme identified recurring interactions among the engineering domains constituting EF-IVIS. The available evidence indicates that decisions made within one domain may constrain or influence activities in other domains, supporting the systems engineering treatment of an intelligent vision inspection system as an interconnected engineering structure rather than a set of independent technological modules.

The principal cross-domain dependencies identified from the combined investigations and supporting engineering literature include:

- Inspection requirements constrain and guide image acquisition, measurement, artificial intelligence, and industrial-integration decisions;
- Image acquisition and illumination engineering are interdependent and jointly influence the information available for subsequent measurement and AI-based analysis;
- Image acquisition and illumination conditions influence the information available for measurement;
- Image acquisition, illumination engineering, and measurement strategy jointly influence the conditions under which AI-based inspection is implemented and evaluated;
- Measurement strategy and AI-based decision-making impose constraints on industrial integration and deployment;
- Operational observations may initiate revision of one or more affected engineering domains.

**Figure 9:**
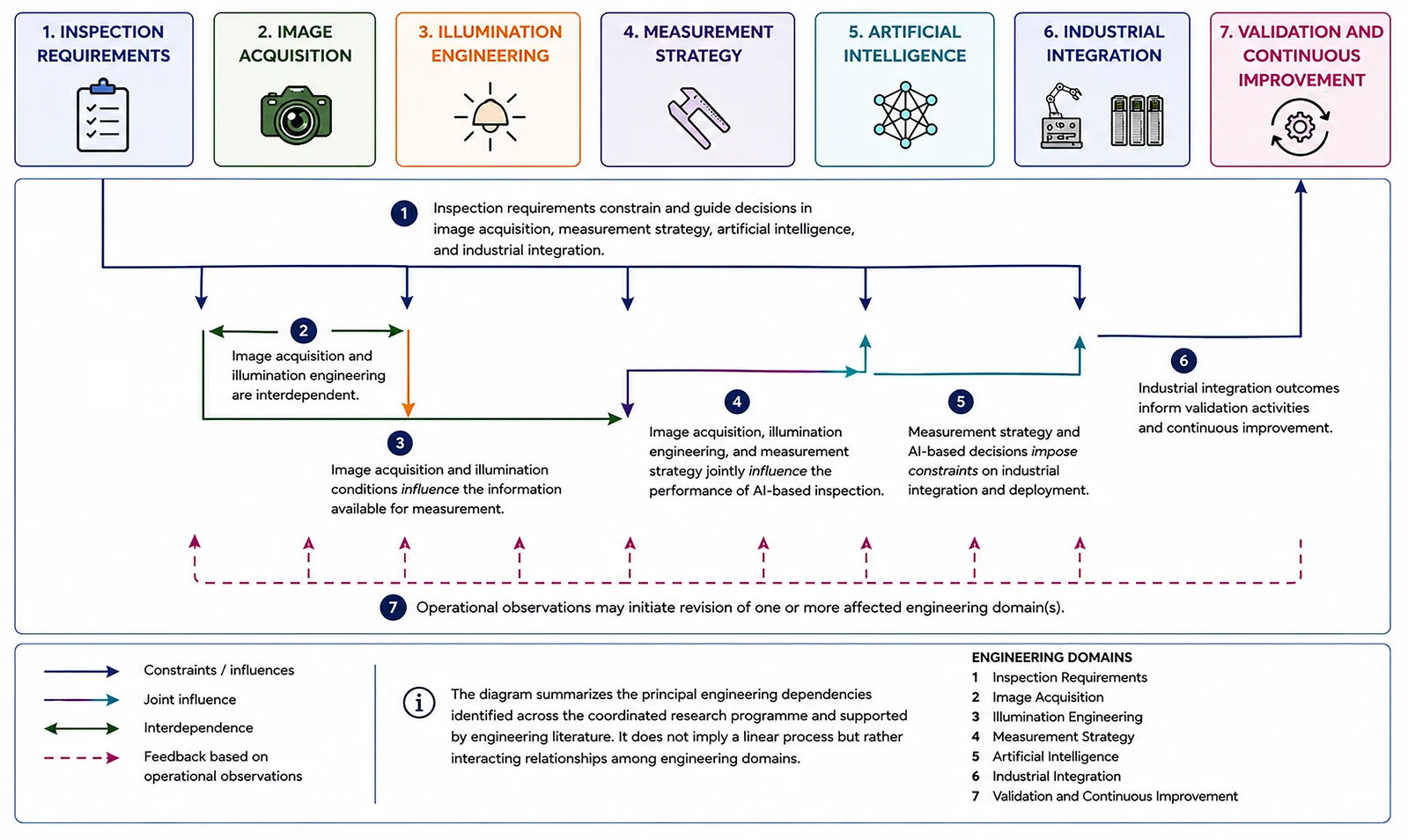
Principal cross-domain engineering dependencies within EF-IVIS.

These relationships are consistent with established systems engineering principles and with the evidence obtained from the coordinated investigations [14,15,16,17,18,19,43,44,45,46]. They support the EF-IVIS proposition that system-level inspection performance depends on the consistency of decisions made across interacting engineering domains rather than solely on the performance of individual technological components. The principal dependencies are summarized schematically in Figure 9, while Table 11 provides their engineering interpretation and supporting evidence.

The dependency analysis indicates that evaluating individual engineering domains in isolation does not capture the cross-domain constraints identified within the coordinated investigations. EF-IVIS therefore emphasizes consistency and traceability among engineering decisions across the development process, while the system-level effectiveness of this approach remains to be evaluated prospectively in complete industrial projects.

### 4.5. Evidence-Based Assessment of EF-IVIS

The available investigations provide differentiated support for EF-IVIS rather than equivalent experimental validation of all framework domains. Direct experimental or industrial evidence supports selected aspects of image acquisition, illumination engineering, measurement strategy, artificial intelligence, and industrial integration, whereas inspection requirements and validation and continuous improvement rely more strongly on cross-study synthesis and established engineering principles.

The evidence supports the internal consistency and practical relevance of the proposed domain structure and cross-domain dependencies. Because the same investigations contributed to both framework development and retrospective assessment, however, independent prospective validation remains necessary. Such validation should apply EF-IVIS throughout a new industrial project using predefined requirements, Decision Gate criteria, system-level indicators, and documented engineering iterations.

## 5. Engineering Design Guidelines for Intelligent Vision Inspection Systems

### 5.1. Engineering Design Philosophy

The Engineering Framework for Intelligent Vision Inspection Systems (EF-IVIS) was developed not only to organize engineering knowledge but also to provide a practical methodology supporting the systematic design of intelligent industrial inspection systems. Consequently, the framework should be interpreted as a decision-support methodology that guides engineers from the initial definition of inspection objectives to the deployment and continuous improvement of industrial inspection systems.

Unlike technology-oriented approaches, which frequently begin with the selection of cameras, sensors, or artificial intelligence algorithms, EF-IVIS recommends adopting a requirement-driven engineering philosophy. This approach is consistent with established systems engineering principles, where functional requirements define subsequent engineering decisions throughout the complete system lifecycle [1,2,3,4,5,6].

The engineering guidelines presented in this section summarize the practical implications of the proposed framework and the industrial evidence supporting its individual technological domains.

### 5.2. Engineering Workflow Recommended by EF-IVIS

Based on the coordinated research programme and the resulting framework, EF-IVIS organizes the development of intelligent vision inspection systems into seven principal engineering stages. Although presented in a logical sequence, these stages may require iteration and feedback between engineering domains as system development progresses.

Stage 1—Define Inspection Requirements

The design process should begin with a comprehensive analysis of manufacturing objectives rather than technological capabilities. Engineers should specify:

- Inspection objectives;
- Critical quality characteristics;
- Dimensional tolerances;
- Production rate;
- Environmental conditions;
- Acceptance criteria.

The completeness and consistency of engineering requirements provide the basis for subsequent engineering decisions and application-specific acceptance criteria [12,13,14,15,16,17,51].

Stage 2—Design Image Acquisition

Image acquisition conditions should be established and verified before downstream inspection methods are finalized. Recommended engineering activities include:

- Sensor selection;
- Lens selection;
- Field-of-view analysis;
- Camera positioning;
- Calibration;
- Verification of image quality.

Camera and image-sensor selection should be supported by standardized characterization procedures defined in EMVA 1288 [59].

The available evidence and machine vision literature indicate that limitations in acquired image information can constrain the performance of subsequent measurement and AI-based inspection methods [26,27,28,29,30,31,36,37,38,39,45].

Stage 3—Optimize Illumination

Illumination should be considered an independent engineering subsystem. The following engineering principles are recommended:

- Maximize feature visibility;
- Minimize reflections;
- Ensure illumination repeatability;
- Maintain optical stability;
- Verify contrast under production conditions.

Illumination conditions should be established and assessed before downstream inspection methods are finalized, because they influence the visual information available for measurement and classification [26,27,28,29,30,31,45].

Stage 4—Select Measurement Strategy

Measurement methodology should be selected according to the inspection objective, required information, and production constraints rather than the availability of particular software tools. Deterministic methods may be appropriate when inspected characteristics can be explicitly represented through dimensional, geometric, or feature-based criteria. Typical applications may include:

- Dimensional verification;
- Inspection of geometrically well-defined features;
- Feature-based evaluation using explicitly configured functions and acceptance limits;
- Applications requiring interpretable quantitative outputs.

AI-assisted methods may be considered when the inspection task involves characteristics that are difficult to represent through fixed deterministic rules, including:

- Visually variable or irregular defects;
- Surface anomalies;
- Complex or variable feature patterns;
- Inspection tasks based on similarity or deviation from learned reference data.

Hybrid inspection strategies may be considered when deterministic and AI-assisted approaches provide complementary information. Investigation [44] supports this complementary interpretation by demonstrating differences in the type of information generated, configuration requirements, processing time, and sensitivity of inspection outputs to object displacement and rotation. However, the performance of a complete hybrid configuration was not independently quantified in the reported investigation.

Selection among deterministic, AI-assisted, and hybrid approaches should therefore consider the required inspection information, feature characteristics, processing-time constraints, robustness under expected operating conditions, interpretability requirements, and, where quantitative metrology is involved, measurement uncertainty and traceability requirements [32,33,34,35,36,37,38,39,40,41,42,43,44,47,48,49,50].

Stage 5—Develop Artificial Intelligence Models

Artificial intelligence should be treated as one component of the overall inspection architecture rather than as a substitute for engineering knowledge. Within EF-IVIS, AI development should remain consistent with the inspection requirements, available image information, measurement strategy, computational architecture, and intended production environment. Engineering activities may include:

- Preparation and verification of representative image datasets;
- Annotation verification where supervised learning is used;
- Definition of regions of interest and relevant preprocessing procedures;
- Selection of an appropriate AI methodology and model architecture;
- Model training and configuration;
- Evaluation of model outputs against application-specific inspection requirements;
- Assessment of robustness to relevant input variability;
- Evaluation of processing time and computational requirements;
- Explainability or interpretability assessment where required by the application;
- Preparation of the model for integration with the industrial inspection system.

Model accuracy should not be treated as the only evaluation criterion. Depending on the application, AI-based inspection should also be assessed with respect to repeatability, robustness to relevant operating variability, computational efficiency, processing time, deployment constraints, and compatibility with the surrounding industrial system [32,33,34,35,36,37,38,39,40,41,42,43]. The numerical acceptance criteria for these characteristics remain application-specific and should be derived from the inspection requirements defined earlier in the EF-IVIS process.

For applications with stringent latency or throughput requirements, hardware acceleration may also be considered as part of the computational architecture. FPGA-based implementations can provide an alternative for selected image-processing, preprocessing, or inference functions where deterministic execution and highly parallel processing are required [60]. Within EF-IVIS, FPGA technology is treated as an application-specific implementation option rather than as a separate engineering domain. Its use should therefore be justified by the timing, throughput, integration, flexibility, and maintainability requirements of the particular inspection system.

Stage 6—Integrate with Manufacturing Systems

Industrial deployment requires verified interaction between the inspection system and the manufacturing infrastructure relevant to the target application. Recommended engineering activities may include:

- PLC integration;
- Communication verification;
- Synchronization;
- MES connectivity;
- OPC UA implementation;
- Verification of deterministic or otherwise application-required communication behaviour.

Industrial communication and control interfaces should be verified before production deployment [1,2,3,4,5,6,9,10,11,20,21,22,23,24,25,46]. Where OPC UA is selected for machine vision integration, implementation should follow the applicable OPC UA for machine vision companion specification [61].

Before production release, the complete inspection system should be evaluated at Decision Gate DG-6. This gate verifies system-level compliance with the original inspection requirements, end-to-end functional performance, measurement and decision reliability under production conditions, communication integrity, operational safety, traceability, and readiness for industrial deployment. Successful completion of DG-6 authorizes transition from system implementation to monitored production operation.

Stage 7—Continuous Improvement

Inspection systems should evolve together with manufacturing processes. Continuous engineering improvement should include:

- Monitoring inspection performance;
- Analysis of false classifications;
- Optimization of illumination;
- Recalibration;
- Retraining AI models;
- Updating engineering requirements.

Continuous engineering improvement is intended to support adaptation to changes in products, manufacturing processes, equipment, environmental conditions, and inspection requirements [1,2,3,4,5,6,9,10,11,14,15,16,17,18,19].

Operational performance should be reviewed periodically and whenever significant changes occur in products, manufacturing processes, equipment, environmental conditions, or inspection requirements. Decision Gate DG-7 evaluates whether the inspection system continues to satisfy the specified performance and quality requirements. The assessment should consider measurement-system drift, false-positive and false-negative rates, model drift or degradation, communication reliability, calibration status, system downtime, and changes in production requirements.

Failure to satisfy the defined criteria should initiate appropriate corrective actions, including recalibration, illumination adjustment, model retraining, revision of engineering requirements, or a return to one or more relevant EF-IVIS engineering domains.

Unlike the preceding gates, DG-7 is a recurring lifecycle review gate rather than a one-time development decision. At the current stage of framework development, DG-7 is conceptually defined and supported by cross-study observations, whereas its long-term effectiveness requires longitudinal industrial validation.

### 5.3. Engineering Decision Matrix

The experimental programme enabled the formulation of practical engineering recommendations supporting decision-making throughout system development. The resulting domain-specific recommendations and their supporting evidence are summarized in Table 12.

### 5.4. Common Engineering Pitfalls

The combined observations from the industrial investigations and the supporting engineering literature indicate several recurring implementation risks that may adversely affect inspection system performance. These include:

- Selection of hardware before defining inspection requirements;
- Inadequate illumination design;
- Insufficient image calibration;
- Inappropriate selection of measurement methodology;
- Overreliance on artificial intelligence without engineering validation;
- Neglect of industrial communication requirements;
- Omission of post-deployment performance monitoring.

Addressing these issues during system development may reduce the likelihood of downstream integration problems and support more systematic risk management during industrial deployment. The principal engineering risks identified within the EF-IVIS development process, together with their potential consequences and recommended mitigation strategies, are summarized in Table 13.

### 5.5. Generalization and Applicability of EF-IVIS

Although the coordinated research programme focused on four industrial investigations, EF-IVIS was formulated at the methodological level rather than around a specific hardware platform, software environment, or manufacturing process. Its engineering domains describe activities and decision relationships that are relevant to a broad class of intelligent vision inspection systems, including requirements definition, image acquisition, illumination, measurement, artificial intelligence, industrial integration, validation, and lifecycle improvement.

The framework is also independent of specific machine vision vendors, artificial intelligence libraries, controller platforms, or communication technologies. This technology-independent formulation is intended to allow the same engineering structure to be instantiated using different implementation technologies while preserving the relationships between requirements, engineering decisions, validation criteria, and lifecycle feedback.

This characteristic suggests potential methodological transferability across different industrial applications. However, the present investigations do not provide sufficient evidence to demonstrate transferability across industrial sectors, organizations, development teams, hardware platforms, or manufacturing environments. Such transferability can only be established through prospective application of EF-IVIS in independent projects using different technologies and operating conditions.

Consequently, the applicability of EF-IVIS beyond the investigated cases should currently be interpreted as a methodological proposition rather than an experimentally demonstrated property. Future studies should evaluate whether independent engineering teams can apply the framework consistently and whether its domain structure, Decision Gates, and lifecycle logic remain useful across different industrial inspection contexts.

## 6. Discussion and Limitations

The preceding sections introduced the architecture of EF-IVIS, examined the experimental and industrial evidence supporting its engineering domains, and translated the resulting observations into practical design guidelines. The present section interprets the principal findings from a systems engineering perspective, clarifies the scope and strength of the available evidence, and discusses the scientific and industrial implications of the proposed framework. Particular attention is given to the distinction between direct experimental and industrial support, cross-cutting retrospective evidence derived from the coordinated research programme, methodological support, and elements of the framework that remain to be validated prospectively.

### 6.1. Interpretation of the Main Findings

The principal finding of this study is that the engineering of an intelligent vision inspection system cannot be reduced to the independent selection or optimization of a camera, inspection algorithm, measurement function, artificial intelligence model, or communication technology. The coordinated investigations indicate that engineering decisions made in different technological domains are interrelated and that their compatibility should therefore be considered at the system level [43,44,45,46]. This interpretation is consistent with established systems engineering principles, which emphasize interactions among system elements, requirements, interfaces, verification activities, and lifecycle processes [14,15,16,17,18,19].

The illumination investigation [45] provides direct evidence for one such dependency. Changes in illumination conditions resulted in different shape- and colour-recognition outcomes under otherwise controlled imaging conditions. White illumination produced the most consistent recognition in the investigated configuration, whereas coloured illumination resulted in application-dependent classification and detection changes. These observations support the distinction between image acquisition and illumination engineering within EF-IVIS while demonstrating that the information available to downstream inspection activities depends on upstream image-formation conditions [26,27,28,29,30,31,45].

Investigation [44] provides complementary evidence concerning measurement strategy. Deterministic and AI-assisted inspection functions differed in the type of information generated, configuration requirements, computational demand, and sensitivity of inspection outputs to changes in object displacement and rotation. The reported results therefore support treating method selection as an engineering decision dependent on the inspection objective and production constraints rather than on an assumed universal hierarchy between deterministic and AI-assisted methods. Hybrid configurations may be considered when the two approaches provide complementary information, although the performance of a complete hybrid strategy was not independently quantified in the reported investigation [44,47,48,49,50].

The artificial intelligence investigation [43] further demonstrates the importance of engineering preparation of input data. ROI definition and masking influenced the reported classification outcome, indicating that AI-based inspection cannot be considered independently of image preparation and the definition of the analysed region. However, the investigation did not provide a comprehensive evaluation of model robustness, generalization, explainability, long-term drift, or other lifecycle characteristics. The evidence therefore supports selected aspects of the artificial intelligence domain rather than comprehensive validation of AI-related Decision Gate criteria [32,33,34,35,36,37,38,39,40,41,42,43].

Investigation [46] extends this system-level interpretation to industrial integration. The implemented camera–PLC architecture provided quantitative evidence concerning communication timing, controller-level verification, throughput, network-load response, and system behaviour under selected operating perturbations. These results demonstrate that the practical assessment of an inspection architecture involves integration characteristics in addition to camera-level inspection performance. At the same time, the investigation was limited to a specific camera, PLC, and communication architecture and therefore does not establish broad interoperability or scalability across arbitrary industrial implementations [1,2,3,4,5,6,9,10,11,20,21,22,23,24,25,46].

Taken together, the investigations provide differentiated evidence for the EF-IVIS proposition that intelligent vision inspection should be engineered as an interconnected system of mutually constrained domains. The evidence does not establish a quantitative causal model linking all seven domains, nor does it constitute independent prospective validation of the complete framework. Rather, it supports the relevance of coordinating requirements, image formation, measurement strategy, AI-based decision-making, industrial integration, and lifecycle feedback within a common systems engineering structure.

### 6.2. Scope and Strength of Experimental Support

The evidence supporting EF-IVIS should be interpreted according to the manner in which the framework was developed. The four investigations were used both as sources of engineering knowledge during the bottom-up synthesis and as retrospective evidence for assessing the relevance of the resulting domains [43,44,45,46]. The study therefore demonstrates the internal consistency and practical grounding of EF-IVIS, but it does not yet constitute fully independent external validation of the complete framework. This distinction is important because direct experimental support is stronger for some domains than for others.

Direct experimental or industrial evidence is available for selected aspects of image acquisition and for the illumination engineering, measurement strategy, artificial intelligence, and industrial integration domains. Inspection requirements and validation and continuous improvement are supported primarily through cross-study synthesis and established systems engineering principles. Table 14 summarizes these differentiated forms of support, the evidence actually reported or observed in the supporting investigations, the principal limitations, and the current status of the corresponding Decision Gates.

Table 14 shows that the empirical basis of EF-IVIS is heterogeneous. Direct evidence is strongest for the technological and integration activities explicitly examined in investigations [43,44,45,46], whereas broader lifecycle and requirements-related elements rely to a greater extent on retrospective synthesis and established engineering principles. The Decision Gates should therefore be interpreted as structured assessment points whose complete criteria and numerical thresholds require application-specific definition and prospective evaluation.

### 6.3. Scientific and Industrial Implications

From a scientific perspective, EF-IVIS shifts the unit of analysis from the performance of an isolated technology toward the engineering coherence of the complete inspection system. Existing machine vision and artificial intelligence studies commonly report domain-specific indicators such as accuracy, precision, recall, processing time, repeatability, or measurement uncertainty. These indicators remain essential for evaluating individual technological solutions, but they do not by themselves describe whether image acquisition, measurement, decision logic, communication, and production integration are mutually compatible. EF-IVIS complements such domain-specific evaluation by providing a structure in which technical indicators are related to engineering requirements, decision criteria, verification activities, and lifecycle feedback.

A second scientific implication concerns the explicit representation of cross-domain dependencies. EF-IVIS makes visible relationships between engineering decisions that are often analysed separately. Inspection requirements constrain acceptable performance and subsequent technology selection; image acquisition and illumination decisions affect the information available for measurement and AI-based analysis; measurement strategy influences the confidence associated with inspection decisions; and manufacturing integration imposes timing, communication, and deployment constraints on the overall architecture. These relationships provide a basis for future research on quantitative dependency models, trade-off analysis, and multi-objective optimization of intelligent inspection architectures. The framework also provides a potential structure for integration with Model-Based Systems Engineering and SysML representations to improve traceability between requirements, system architecture, verification evidence, and operational observations [14,15,16,17,18,19,43,44,45,46,53].

From an industrial perspective, EF-IVIS is intended to function as a structured decision-support framework rather than as a fixed technological recipe. It encourages engineering teams to define measurable inspection objectives before technology selection, verify the adequacy of acquired image information before downstream algorithm development, select measurement and AI methods according to the inspection objective and production constraints, and assess industrial communication and integration before production deployment. The Decision Gates provide explicit assessment points at which the outputs of one engineering domain can be reviewed before progression to subsequent development activities.

By structuring these assessment points, EF-IVIS is intended to support earlier identification of unresolved technical issues, improve documentation and decision traceability, and potentially reduce the likelihood of late-stage redesign. These potential benefits have not yet been quantified through prospective comparative studies and should therefore not be interpreted as demonstrated improvements in engineering efficiency, implementation cost, or project risk.

The framework may also facilitate collaboration among specialists who use different terminology, models, and performance criteria. Machine vision engineers, metrologists, AI developers, automation engineers, quality personnel, and production specialists can relate their activities to a common sequence of requirements, engineering decisions, outputs, and assessment criteria. Such coordination may be particularly useful in multidisciplinary projects where optimization within one engineering domain introduces constraints or failure modes in another.

The broader applicability of EF-IVIS should nevertheless be interpreted cautiously. Its technology-independent structure suggests potential methodological transferability across different inspection applications and implementation technologies, but the present investigations do not demonstrate equivalent usability or effectiveness across industrial sectors, organizations, development teams, or hardware platforms. Applications in safety-critical, highly regulated, exceptionally high-speed, or otherwise specialized environments may additionally require domain-specific engineering processes, standards, verification procedures, and acceptance criteria. Prospective application of EF-IVIS in independent industrial projects is therefore required to assess its usability, repeatability, transferability, and engineering effectiveness.

### 6.4. Limitations of the Study

Several limitations should be considered when interpreting the results. First, the same four investigations contributed to the development of EF-IVIS and to the retrospective assessment of its engineering domains. This approach is appropriate for bottom-up framework construction, but it creates a degree of methodological dependence between framework generation and retrospective framework assessment. The present results therefore establish internal coherence and industrial relevance rather than independent confirmation of predictive or prescriptive effectiveness.

Second, the investigations were designed for different industrial objectives and employed different technologies, datasets, performance indicators, and experimental procedures [43,44,45,46]. Their heterogeneity is valuable for knowledge synthesis but prevents direct aggregation into a single quantitative measure of framework effectiveness. The current study cannot therefore report a unified improvement percentage, common effect size, or statistically pooled comparison between development with and without EF-IVIS. Future validation should define common system-level indicators, such as requirement fulfilment, redesign effort, commissioning time, decision reliability, downtime, maintainability, and lifecycle cost.

Third, the Decision Gates use application-specific acceptance criteria rather than universal numerical thresholds. The worked DG-5 example demonstrates how predefined quantitative limits can be associated with measured evidence and used in a reproducible PASS/FAIL–corrective-action–re-evaluation sequence. However, EF-IVIS does not prescribe universal values for metrics such as contrast, signal-to-noise ratio, measurement uncertainty, model accuracy, false-positive rate, communication latency, throughput, or process capability, because acceptable values depend on the product, production process, operating environment, regulatory context, and manufacturing objectives. Moreover, formal methods for weighting non-mandatory criteria and resolving trade-offs between competing objectives have not yet been developed. Quantitative gate models and trade-off procedures therefore remain an important direction for future research.

Fourth, the continuous improvement component has not been evaluated through a controlled longitudinal study covering product changes, model drift, equipment ageing, recalibration, retraining, and evolving quality requirements over an extended production period. The current evidence supports the need for feedback-driven adaptation, but it does not quantify the long-term benefits or costs of the proposed lifecycle process. Similarly, the framework has not yet been prospectively applied by an independent engineering team to a new system from initial requirements definition to operational validation.

Finally, the industrial investigations cover representative but limited application contexts. Additional studies are needed across sectors with different regulatory, environmental, throughput, and traceability requirements. Direct comparison with alternative engineering approaches under the same project conditions would also be valuable. Such comparison should determine whether EF-IVIS improves engineering outcomes relative to conventional technology-driven development, informal expert practice, or general systems engineering processes that have not been specialized for intelligent vision inspection.

### 6.5. Relation to Existing Engineering Approaches

EF-IVIS is complementary to established systems engineering, machine vision, metrology, AI, automation, and industrial communication methods. These approaches remain necessary for implementing individual engineering activities. EF-IVIS instead defines an IVIS-specific structure through which their outputs, dependencies, requirements, and assessment criteria can be coordinated across the inspection system lifecycle. The more detailed methodological comparison is provided in Section 3.6 and Table 5.

Further development should focus on prospective application in independent industrial projects, comparative assessment against alternative development approaches, formalization of quantitative Decision Gate criteria, and integration with MBSE/SysML. Such investigations are required to determine whether EF-IVIS improves engineering outcomes and which framework elements can be generalized across industrial sectors and technology platforms.

## 7. Conclusions and Future Research Directions

The increasing complexity of industrial vision inspection requires engineering approaches that extend beyond the independent optimization of cameras, image-processing algorithms, measurement functions, artificial intelligence models, or communication components. This study introduced the Engineering Framework for Intelligent Vision Inspection Systems (EF-IVIS), a domain-specific systems engineering approach intended to coordinate the development of intelligent industrial vision inspection systems. EF-IVIS does not introduce new fundamental systems engineering principles. Instead, it operationalizes established principles for the specific technological and operational conditions of intelligent vision inspection.

EF-IVIS organizes engineering activities into seven interconnected domains: inspection requirements, image acquisition, illumination engineering, measurement strategy, artificial intelligence, industrial integration, and validation and continuous improvement. The framework explicitly represents cross-domain dependencies, engineering outputs, assessment criteria, Decision Gates, and lifecycle feedback while remaining independent of particular cameras, software platforms, AI models, industrial controllers, or communication technologies. Its principal methodological contribution is therefore the coordinated organization of engineering decisions that are frequently treated separately in conventional machine vision development.

The framework was developed through a bottom-up synthesis of established engineering principles and four complementary industrial investigations addressing AI-based defect detection, measurement strategies, illumination conditions, and PLC-supported industrial integration [43,44,45,46]. The available evidence is intentionally interpreted in a differentiated form. Direct experimental or industrial evidence supports selected aspects of image acquisition and the illumination engineering, measurement strategy, artificial intelligence, and industrial integration domains. Inspection requirements and validation and continuous improvement are supported primarily through cross-study synthesis and established systems engineering principles. Consequently, the present work provides empirical grounding and retrospective support for the relevance and internal consistency of the EF-IVIS structure rather than independent prospective validation of the complete framework.

The Decision Gates provide explicit assessment points for relating domain outputs to application-specific acceptance requirements. The retrospective DG-5 example demonstrates how industrial implementation evidence can be translated into engineering assessment criteria and gate decisions. The numerical values reported in the supporting investigation are not universal EF-IVIS thresholds, and prospective applications must define acceptance limits from the inspection requirements and production constraints of the specific system. Quantitative gate models, trade-off procedures, and traceability mechanisms therefore remain important subjects for further development.

Prospective validation should apply EF-IVIS throughout the complete lifecycle of newly developed industrial inspection systems, preferably through independent engineering teams and comparative studies. Such investigations should evaluate usability, repeatability, transferability, engineering effort, commissioning performance, decision reliability, maintainability, lifecycle cost, and the practical effectiveness of the Decision Gates across different manufacturing sectors, hardware platforms, inspection objectives, production rates, and environmental conditions. This work is necessary to determine which elements of EF-IVIS can be generalized and which require application-specific adaptation.

Future development may also investigate integration with Model-Based Systems Engineering and SysML to strengthen traceability between requirements, architecture, verification evidence, and operational observations [14,15,16,17,18,19,53]. Integration with model-driven and synthesis-based approaches for cyber-physical production systems may support partial automation of transitions between requirements, architecture definition, implementation, and verification. Additional research directions include Digital Twin technologies, adaptive inspection, Edge AI, hardware-accelerated computational architectures for time-critical applications, foundation models, and increasingly autonomous manufacturing systems. Long-term industrial studies are additionally required to evaluate the validation and continuous improvement domain under changing products, process conditions, equipment states, environmental conditions, and quality requirements.

In conclusion, EF-IVIS provides a structured domain-specific systems engineering approach whose architecture is grounded in established engineering principles and informed by evidence from complementary industrial vision-inspection investigations. The current study supports the internal consistency and practical relevance of its engineering domains and cross-domain relationships, while the effectiveness, usability, repeatability, transferability, and complete lifecycle performance of EF-IVIS remain to be established through independent prospective validation.

## Figures and Tables

**Table 1:** Evolution of industrial vision inspection systems from the perspective of engineering complexity.

Engineering Dimension	Stage 1 Manual Inspection (Before 1990s)	Stage 2 Conventional Machine Vision (1990s–Early 2000s)	Stage 3 Advanced Vision Systems (2000s–Early 2010s)	Stage 4 Intelligent Vision Systems (2010s–Early 2020s)	Stage 5 Autonomous Quality Engineering (2020s and Beyond)
Inspection Objective	Defect detection after production	Automated defect detection and measurement	High-accuracy measurement and process monitoring	Real-time quality assurance and process control	Autonomous quality engineering and self-optimizing production
Sensing & Image Acquisition	Human visual observation	Monochrome/colour cameras, fixed lighting	High-resolution sensors, optimized optics	Multi-sensor fusion, adaptive illumination	Context-aware sensing, self-calibration
Data Processing & Measurement	Subjective judgement, no measurements	Rule-based algorithms, 2D measurements	Advanced algorithms, 2D/3D measurements	AI-based analysis, robust measurements	AI-driven reasoning, uncertainty-aware decisions
Intelligence & Decision-making	Human decision only	Deterministic decision rules	Statistical analysis, pattern recognition	Machine learning, deep learning	Autonomous decision-making, continuous learning
System Integration & Automation	No integration	Standalone systems, manual operation	Integration with PLC, simple feedback	Full automation, closed-loop control	Autonomous operation across cyber-physical systems
Communication & Data Management	No data recording	Local data storage	Industrial networks, data logging	Real-time communication, edge computing	IIoT connectivity, cloud-edge collaboration
Validation & Verification	None	Basic functional test	Accuracy and repeatability evaluation	Robustness and reliability evaluation	Continuous validation, self-diagnosis
Maintainability & Flexibility	Depends on operator	Low flexibility, operator dependent	Moderate flexibility, reconfiguration possible	High flexibility, remote maintenance	Self-adaptive systems, predictive maintenance

Note: The table presents a qualitative synthesis of the evolution of industrial vision inspection systems based on the literature [1,2,3,4,5,6,9,10,11]. The stages describe changes in technological capabilities and engineering scope and should not be interpreted as a quantitative maturity, performance, or value-assessment scale.

**Table 2:** Engineering domains investigated within the coordinated research programme.

Experimental Investigation	Engineering Domain	Engineering Objective	Contribution to EF-IVIS
Deep learning defect detection	Artificial intelligence	Adaptive defect recognition	Support for artificial intelligence domain
Measurement methodology	Industrial metrology	Comparison of deterministic and AI-assisted inspection functions	Support for measurement strategy domain
Illumination engineering	Image acquisition	Illumination-dependent shape and colour recognition	Support for image acquisition domain
PLC integration	Industrial automation	Real-time production integration	Support for industrial integration domain

**Table 3:** General engineering structure of EF-IVIS.

Framework Element	Description	Primary Objective
Engineering Inputs	Functional and technical information entering the current engineering domain	Define design constraints
Engineering Activities	Analysis, modelling, optimization, configuration and implementation	Develop engineering solution
Engineering Decisions	Selection of engineering alternatives based on defined criteria	Support systematic decision-making
Engineering Outputs	Information transferred to subsequent engineering domains	Provide assessed engineering outputs
Validation Criteria	Assessment of engineering outputs against defined criteria	Assess compliance with defined criteria
Engineering Feedback	Iterative refinement based on assessment results	Support iterative refinement

**Table 4:** Evidence supporting the engineering domains of EF-IVIS.

Engineering Domain	Literature Support	Research Evidence	Type of Support
Inspection requirements	Systems engineering and requirements engineering [12,13,14,15,16,17,18,19,51,52]	All investigations [43,44,45,46]	Cross-cutting and retrospective
Image acquisition	Machine vision and image formation [26,27,28,29,30,31]	Illumination investigation [45]	Direct experimental
Illumination engineering	Machine vision, optical inspection, and industrial metrology [26,27,28,29,30,31,47,48,49,50]	Illumination investigation [45]	Direct experimental
Measurement strategy	Industrial metrology and measurement uncertainty [47,48,49,50]	Measurement investigation [44]	Direct experimental
Artificial intelligence	Artificial intelligence for industrial inspection [32,33,34,35,36,37,38,39,40,41,42]	AI-based defect-detection investigation [43]	Direct experimental
Industrial integration	Industrial automation, communication, and cyber-physical production systems [1,2,3,4,5,6,9,10,11,20,21,22,23,24,25]	PLC integration investigation [46]	Direct industrial
Validation and Continuous Improvement	Systems engineering, quality management, and Smart Manufacturing [1,2,3,4,5,6,9,10,11,12,13,14,15,16,17,18,19,51,52]	Combined research programme [43,44,45,46]	Integrated, cross-domain, and retrospective

**Table 5:** Positioning of EF-IVIS relative to established systems engineering and machine vision development approaches.

Methodological Approach	Primary Scope	IVIS-Specific Engineering Structure	Cross-Domain Integration Relevant to IVIS	Decision and Assessment Logic	Relationship to EF-IVIS
General systems engineering and lifecycle approaches [14,15,17,18]	System lifecycle processes, stakeholder needs, architecture, implementation, verification, validation, operation, and evolution	Domain-independent; no prescribed structure specific to intelligent vision inspection	Provides general mechanisms for coordinating multiple engineering disciplines, but does not prescribe the specific interaction of vision, illumination, metrology, AI, and industrial automation	Supports lifecycle reviews and engineering decision processes, but does not define IVIS-specific Decision Gates	Provides the general systems engineering foundation on which EF-IVIS is based
Requirements engineering [16]	Definition, analysis, specification, validation, and management of system requirements throughout the lifecycle	Focused on requirements rather than the complete IVIS engineering architecture	Establishes requirements and traceability that can constrain downstream engineering domains	Provides requirements verification and validation processes, but no IVIS-specific cross-domain gate sequence	Provides the methodological basis for the EF-IVIS inspection requirements domain
MBSE/SysML [19]	Model-based representation of system requirements, structure, behaviour, interfaces, and verification relationships	General-purpose and tailorable; does not prescribe an IVIS-specific domain structure	Can represent cross-domain relationships, including vision, metrology, AI, and automation, but does not define which dependencies are required for IVIS	Can represent verification criteria and decision information, but does not prescribe DG-1–DG-7 or equivalent IVIS-specific gates	Provides a potential formal modelling environment for representing and extending EF-IVIS
Architecture-description approach, ISO/IEC/IEEE 42010 [53]	Structured description of system architectures, viewpoints, views, and model kinds	Domain-independent; concerned with architecture description rather than an IVIS development methodology	Allows multidisciplinary architectural concerns to be represented through viewpoints, but does not prescribe the specific IVIS engineering domains or their lifecycle dependencies	Does not prescribe engineering lifecycle processes or application-specific acceptance gates	Can support formal architectural description of an EF-IVIS-based system
Machine vision development framework, Zhu et al. [54]	Reusable software framework for industrial product-appearance inspection	Machine vision specific, with modules for image acquisition, configuration, database management, GUI, I/O communication, and execution architecture	Integrates principal software components of an industrial vision application; its primary scope is software and implementation architecture rather than multidisciplinary lifecycle coordination	Provides software workflow and implementation structure rather than a lifecycle sequence of systems engineering Decision Gates	Represents a direct domain-specific comparator; EF-IVIS extends the scope from vision-software development to multidisciplinary systems engineering
VDI/VDE/VDMA 2632 series [55,56]	Machine vision requirement/system specification and acceptance assessment	Directly machine vision specific, particularly for specification and acceptance activities	Addresses machine vision influencing factors and performance requirements, but does not prescribe a complete lifecycle integrating metrology, AI, industrial communication, and automation	Provides requirement/specification and acceptance procedures, including classification-performance assessment, but not a seven-domain cross-domain gate sequence	Provides domain-specific specification and acceptance principles that complement EF-IVIS and can support the inspection requirements and validation criteria used at its Decision Gates
Conceptual framework for machine vision integration in manufacturing SMEs, Werheid et al. [57]	Requirement-driven integration of machine vision in manufacturing SMEs	Machine vision-specific workflow comprising requirements and specifications, hardware setup, software setup, image analysis, and operational integration	Integrates technical and organizational aspects of machine vision deployment and includes iterative backward loops	Includes conditional decision nodes and iterative workflow logic, but does not define evidence-based acceptance gates between IVIS engineering domains	Represents a closely related domain-specific integration framework; EF-IVIS differs primarily through its seven-domain IVIS structure, explicit cross-domain dependency model, dedicated treatment of metrology and AI, and requirements-derived DG-1–DG-7 assessment sequence
EF-IVIS	Domain-specific systems engineering of intelligent industrial vision inspection from requirements to operational improvement	Seven interconnected domains: inspection requirements, image acquisition, illumination engineering, measurement strategy, artificial intelligence, industrial integration, and validation and continuous improvement	Explicitly coordinates image formation, illumination, metrology, AI, industrial communication, automation, and lifecycle validation, including identified cross-domain dependencies	Introduces DG-1–DG-7 as explicit assessment points between successive engineering stages; quantitative thresholds remain application-dependent	Domain-specific operationalization of established systems engineering principles for intelligent vision inspection, retrospectively grounded in investigations [43,44,45,46]

**Table 6:** Evidence mapping between the industrial investigations and the engineering domains of EF-IVIS.

Evidence Source	EF-IVIS Engineering Domain	Type of Support
All investigations [43,44,45,46] + systems engineering and requirements engineering principles [12,13,14,15,16,17,18,19]	Inspection requirements	Cross-cutting, retrospective, and methodological
Illumination investigation [45]	Image acquisition	Direct experimental
Illumination investigation [45]	Illumination engineering	Direct experimental
Measurement investigation [44]	Measurement strategy	Direct experimental
AI-based defect-detection investigation [43]	Artificial intelligence	Direct experimental
PLC integration investigation [46]	Industrial integration	Direct industrial
Combined research programme [43,44,45,46] + systems engineering and quality management principles [12,13,14,15,16,17,18,19]	Validation and continuous improvement	Integrated, cross-domain, retrospective, and methodological

**Table 7:** Empirical evidence provided by the industrial investigations supporting EF-IVIS.

Investigation	Experimental Setup and Scope	Dataset/Test Scope	Reported Indicators and Variability	Principal Reported Results	EF-IVIS Proposition Supported
AI-based defect detection [43]	Cognex In-Sight D900 smart camera with illuminator; inspection of the machined surface of an automotive suspension knuckle; ViDiDetect Red Analyze in unsupervised mode; ROI definition and masking for burr, chip, and tool-wear detection	500 labelled images; 80% training, approximately 10% validation and 10% testing; an additional post-mask assessment was reported	Defect-area score and Pass/Fail classification; classification thresholds expressed in mm^2^. Accuracy, precision, recall, F1-score, and statistical uncertainty were not reported.	ROI masking reduced ambiguous classification outcomes by excluding irrelevant image regions. The post-mask analysis reported classification limits of 6.32 mm^2^ for good classification and 6.65 mm^2^ for bad classification.	AI-based inspection depends on appropriate dataset preparation, ROI definition, masking, and upstream image formation; supports the artificial intelligence domain and its dependency on acquisition-related decisions
Measurement methodology [44]	Cognex D900 vision system; comparison of deterministic user-configured measurement and inspection functions with ViDiDetect and ViDiRead deep-learning tools	ViDi training dataset of 24 images; 50% of the prepared images recommended for learning; separate stability evaluation performed on 73 images with changes in object displacement and rotation	Defect-score separation, processing time, and sensitivity of inspection-function outputs to object displacement and rotation. No formal measurement-uncertainty budget was reported.	The reported difference between good and bad ViDi classification results was 0.56 mm^2^ within the 4.8–6.4 mm^2^ range. AI-based processing required 227–262 ms, whereas deterministic functions required approximately 30–200 ms depending on the inspected feature. Function outputs changed with object displacement and rotation.	Measurement and inspection strategy should be selected according to the required information, robustness, adaptability, and processing constraints; supports complementary use of deterministic and AI-assisted methods
Illumination engineering [45]	Cognex colour vision system; fixed imaging geometry; shape and colour recognition under white, red, green, and blue illumination	Four object colours investigated under four illumination conditions and two object arrangements; the number of repeated observations was not reported	Correctness of shape and colour recognition under different illumination conditions. No accuracy percentage, standard deviation, repeatability statistic, or measurement uncertainty was reported.	White illumination produced the most consistent shape and colour recognition. Under red illumination, the green object was classified as red. Under green illumination, detection was primarily limited to green and occasionally yellow objects, while the orange object was not always correctly detected. Under blue illumination, detected objects were classified as green, and the orange object was not detected.	Illumination conditions directly affect feature visibility and classification reliability; provides direct experimental evidence for illumination engineering and supporting evidence for image acquisition
PLC-supported industrial integration [46]	Cognex In-Sight 2802C smart camera integrated with an Allen-Bradley Compact GuardLogix PLC through EtherNet/IP implicit cyclic communication; three inspection cases involving 3D-printed holders, automotive electrical connectors, and fibre-optic tubes; PLC-level triple verification	250 3D-printed holders, 300 automotive connector samples, and 600 fibre-optic tubes; a 70/30 training/testing split was reported	Accuracy, precision, recall, F1-score, false-positive rate, end-to-end latency, throughput, network-load sensitivity, and robustness under environmental perturbations.	Accuracy was 96.2%, 95.8%, and 95.2% for the three cases, with F1-scores of 96.1%, 95.7%, and 95.2%, respectively. PLC-level verification reduced the false-positive rate from 7.5% to 5.4%, corresponding to a 28% relative reduction. Mean latency was 54.7 ± 6.3 ms for the simpler inspection task and 58.9 ± 8.1 ms for the more complex classification task. Throughput was approximately 700–950 parts/min. Under ±20% ambient-illumination variation and minor dust deposition, accuracy remained above 93%.	Industrial integration influences end-to-end reliability, communication timing, synchronization, decision verification, and robustness; provides direct industrial evidence for the industrial integration domain

**Table 8:** Engineering influence of image acquisition and illumination within EF-IVIS.

Engineering Activity	Primary Engineering Objective	Potential Downstream Influence	Supporting Evidence
Camera and sensor selection	Required spatial and radiometric image information	Feature detectability and measurement capability	[26,27,28,29,30,31]
Optical configuration	Appropriate field of view, focus, and image geometry	Geometric reliability and feature representation	[26,27,28,29,30,31,47,48,49,50]
Illumination engineering	Stable visibility and discrimination of relevant features	Measurement and classification reliability	[26,27,28,29,30,31,45]
Calibration and image-quality verification	Consistency and traceability of acquired image information	Measurement reliability and downstream validation	[26,27,28,29,30,31,47,48,49,50]

**Table 9:** Evidence-based comparison of measurement and inspection strategies considered within EF-IVIS based on investigation [44].

Engineering Aspect	Deterministic Approach	AI-Assisted Approach	Hybrid Strategy
Type of inspection output	Provides explicitly configured measurements or feature-specific inspection results	Provides deviation or similarity-based assessment without necessarily identifying a specific dimensional cause	Combines explicit dimensional or feature-based information with broader anomaly detection
Definition of inspected features	Inspection features, measurement functions, and acceptance limits are explicitly configured	Relevant patterns are learned from reference images, and deviations are identified from the learned representation	Critical dimensions or known features are configured explicitly, while AI is used to identify additional or unforeseen deviations
Training and configuration effort	Does not require an image-training dataset; inspection limits can be modified directly	Requires preparation and labelling of training images; the reported ViDi experiment used 24 images, with 50% recommended for learning	Requires both deterministic configuration and preparation of AI training data
Processing time in the reported investigation	Approximately 30–200 ms, depending on the inspected feature and configuration	227–262 ms	Not independently quantified in [44]
Defect discrimination reported for the AI case	Not directly comparable because deterministic tools return predefined measurements or inspection results	A 0.56 mm^2^ separation between reported good and bad results within the 4.8–6.4 mm^2^ range was observed	Not independently quantified
Sensitivity to object position and orientation	Inspection-function outputs were affected by displacement and rotation in the reported stability experiment	Position and orientation must be controlled through ROI and fixture handling	Requires verification of robustness for the combined inspection configuration
Principal engineering use	Well-defined dimensions, known features, and tasks requiring explicit interpretable outputs	Detection of broader deviations and defects that may be difficult to define through fixed inspection functions	Combination of deterministic measurement of critical features with AI-based detection of additional anomalies
Main trade-off identified in the study	Greater direct control over inspection functions and lower computational demand	Greater adaptability to visual deviations at the cost of training requirements and longer processing time	Broader inspection capability, but with increased system and configuration complexity

**Table 10:** Worked example of Decision Gate DG-5 using industrial evidence from investigation [46].

DG-5 Criterion	Predefined Application-Specific Acceptance Requirement	Initial Evidence/Measured Result	Initial Assessment	Corrective Action	Re-Evaluated Result
Interface readiness	Inspection, trigger, status, and decision data shall be exchanged through the intended camera–PLC interface without unresolved communication or interface errors	Cognex In-Sight 2802C successfully integrated with Compact GuardLogix through EtherNet/IP implicit cyclic communication	PASS	Not required	—
End-to-end timing	Mean end-to-end response time ≤ 70 ms	54.7 ± 6.3 ms for the simpler task and 58.9 ± 8.1 ms for the more complex classification task	PASS	Not required	—
Network-load robustness	Increase in latency at 50% network-bandwidth saturation ≤ 10%	Latency increase < 6%	PASS	Not required	—
Decision reliability	False-positive rate ≤ 6.0%	Camera-only baseline false-positive rate = 7.5%	FAIL	PLC-level triple-verification logic introduced	False-positive rate = 5.4%, corresponding to a 28% relative reduction
Production throughput	≥700 parts/min for the short-cycle operations considered in the application	Approximately 700–950 parts/min	PASS	Not required	—
Environmental robustness	Classification accuracy ≥93% under ±20% ambient-illumination variation and minor dust deposition	Accuracy remained >93%; false positives increased by approximately 2–3%	PASS	Not required for the defined acceptance criterion; additional shielding/gain-control measures may be considered for more severe conditions	—
Interoperability/scalability	Not mandatory for this application; verification required only for the specified Cognex–Compact GuardLogix–EtherNet/IP architecture	Only one camera platform, PLC architecture, and communication protocol were investigated	N/A	Additional verification required if the deployment architecture is changed	—

**Table 11:** Cross-domain evidence and dependency matrix of EF-IVIS.

Engineering Domain	Primary Dependencies	Principal Engineering Impact	Supporting Evidence
Inspection Requirements	Manufacturing objectives, product characteristics, quality criteria, and production constraints	Definition of system requirements and application-specific acceptance criteria	Cross-study synthesis [43,44,45,46]; systems engineering [14,15,16,17,18,19]
Image Acquisition	inspection requirements and illumination conditions	Availability, quality, and stability of image information for subsequent analysis	Investigations [43,45]; the machine vision literature [26,27,28,29,30,31]
Illumination Engineering	inspection requirements and image acquisition	Feature visibility, contrast, and consistency of acquired image information	Illumination investigation [45]; the machine vision literature [26,27,28,29,30,31]
Measurement Strategy	inspection requirements, image acquisition, and illumination engineering	Selection and reliability of measurement or feature-extraction results used for inspection decisions	Measurement investigation [44]; the industrial-metrology literature [47,48,49,50]
Artificial Intelligence	inspection requirements, image acquisition, illumination engineering, and measurement strategy	Suitability of AI-based decision-making and dependence on image-data preparation and inspection conditions	AI-based defect-detection investigation [43]; the AI literature [32,33,34,35,36,37,38,39,40,41,42]
Industrial Integration	inspection requirements, measurement strategy, artificial intelligence, and communication architecture	Communication timing, synchronization, controller-level decision handling, and production integration	PLC-integration investigation [46]; the industrial communication literature [1,2,3,4,5,6,9,10,11,20,21,22,23,24,25]
Validation and Continuous Improvement	All engineering domains and operational feedback	Monitoring, revalidation, feedback to affected domains, and lifecycle-oriented engineering updates	Cross-study synthesis [43,44,45,46]; systems engineering and Smart Manufacturing [1,2,3,4,5,6,9,10,11,14,15,16,17,18,19]

**Table 12:** Engineering recommendations for the design of intelligent vision inspection systems.

Engineering Domain	Primary Recommendation	Supporting Evidence
Inspection requirements	Define engineering objectives before technology selection	[14,15,16,17,18,19,43,44,45,46]
Image acquisition	Establish and verify image acquisition conditions before finalizing downstream inspection methods	[26,27,28,29,30,31,43,45]
Illumination	Treat lighting as an engineering subsystem	[26,27,28,29,30,31,45]
Measurement	Select methodology according to inspection objectives	[44,47,48,49,50]
Artificial intelligence	Select deterministic, AI-assisted, or hybrid methods according to inspection requirements	[32,33,34,35,36,37,38,39,40,41,42,43]
Industrial integration	Validate communication before deployment	[1,2,3,4,5,6,9,10,11,20,21,22,23,24,25,46]
Continuous improvement	Implement operational monitoring, feedback, and revalidation where changes require engineering intervention	[1,2,3,4,5,6,9,10,11,14,15,16,17,18,19,43,44,45,46]

**Table 13:** Typical engineering risks and recommended mitigation strategies.

Engineering Risk	Potential Engineering Consequence	Recommended Mitigation
Incomplete requirements	Ineffective inspection concept	Formal requirements engineering
Poor illumination	Reduced detection accuracy	Optical optimization and validation
Inadequate calibration	Measurement uncertainty	Calibration and periodic verification
Unbalanced AI dataset	Potential reduction in model generalization	Representative data collection
Communication failures	Production interruptions	Deterministic industrial protocols
Lack of continuous monitoring	Delayed detection of performance degradation	Continuous engineering improvement

**Table 14:** Consolidated evidence basis, reported observations, limitations, and Decision Gate status across the EF-IVIS engineering domains.

EF-IVIS Engineering Domain	Evidence Basis and Scope	Evidence Reported or Observed in Supporting Investigation(s)	Level of Support	Principal Limitation	Decision Gate Status
Inspection Requirements	Cross-study synthesis of investigations [43,44,45,46], supported by systems and requirements engineering principles [12,13,14,15,16,17,18,19]	All four investigations originated from defined inspection objectives, product characteristics, acceptance conditions, or implementation constraints. No dedicated requirements engineering experiment was performed.	Cross-cutting, retrospective, and methodological	Requirements completeness, traceability, and change management have not been evaluated prospectively throughout a new industrial project.	DG-1: Conceptually defined and retrospectively supported; prospective validation remains required.
Image Acquisition	Supporting experimental observations from [43,45] together with machine vision literature [26,27,28,29,30,31]	Investigation [43] included ROI definition, masking, and preparation of image data. Investigation [45] used controlled imaging geometry, exposure, gain, and white-balance settings while evaluating illumination-dependent inspection behaviour.	Direct experimental support for selected acquisition-related conditions, supplemented by the methodological literature	Camera type, lens selection, spatial resolution, calibration strategy, and broader environmental conditions were not independently investigated across the supporting studies.	DG-2: Selected acquisition-related criteria are experimentally supported; quantitative acceptance thresholds remain application-specific.
Illumination Engineering	Illumination investigation [45], supported by the machine vision literature [26,27,28,29,30,31]	Shape and colour recognition were compared under white, red, green, and blue illumination. Illumination-dependent changes in detection and classification outcomes were observed.	Direct experimental	The study did not report a statistical performance dataset, repeatability statistics, measurement uncertainty, or evaluation across a broad range of industrial lighting configurations.	DG-2: Illumination-related assessment is experimentally supported; application-specific acceptance criteria remain necessary.
Measurement Strategy	Measurement investigation [44], supported by industrial-metrology principles [47,48,49,50]	Deterministic and AI-assisted functions were compared with respect to inspection output, configuration, processing time, and sensitivity to object displacement and rotation. AI processing of 227–262 ms and deterministic processing of approximately 30–200 ms were reported; a 73-image stability experiment was also performed.	Direct experimental	No formal measurement-uncertainty budget was reported; broader feature classes, traceability chains, method reproducibility, and complete hybrid configurations require additional evaluation.	DG-3: Selected method-selection, processing-time, and stability criteria are experimentally supported; uncertainty, repeatability, and traceability limits remain application-specific.
Artificial Intelligence	AI-based defect-detection investigation [43], supported by the industrial-AI literature [32,33,34,35,36,37,38,39,40,41,42]	A 500-image dataset was used with ViDiDetect Red Analyze; ROI definition and masking influenced classification outcomes. Post-mask classification limits of 6.32 mm^2^ and 6.65 mm^2^ were reported.	Direct experimental	Accuracy, precision, recall, F1-score, statistical uncertainty, independent-dataset robustness, model drift, and long-term deployment behaviour were not reported.	DG-4: Selected dataset-preparation, ROI, masking, scoring, and classification aspects are experimentally supported; comprehensive gate validation remains required.
Industrial Integration	PLC-supported industrial integration investigation [46], supported by the industrial automation and communication literature [1,2,3,4,5,6,9,10,11,20,21,22,23,24,25]	Cognex In-Sight 2802C and Compact GuardLogix were integrated through EtherNet/IP. Reported evidence included latency, throughput, PLC-level verification, network-load response, and selected environmental perturbations.	Direct industrial	One camera platform, one PLC architecture, and one communication protocol were evaluated; broad multi-vendor interoperability, cybersecurity, arbitrary scalability, and long-term production reliability remain unverified.	DG-5: Operationalized retrospectively using [46]; reported values provide gate evidence but not universal acceptance thresholds.
Validation and Continuous Improvement	Cross-study synthesis of [43,44,45,46], supported by systems engineering, quality management, and Smart Manufacturing principles [1,2,3,4,5,6,7,8,9,10,11,12,13,14,15,16,17,18,19,51]	The investigations contained verification, adjustment, performance-assessment, and feedback activities that provide retrospective evidence for cross-domain review and engineering iteration; no unified lifecycle metric was evaluated.	Integrated, cross-domain, retrospective, and methodological	The complete lifecycle process has not been applied prospectively from requirements definition through long-term operation.	DG-6: Selected system-level verification aspects are retrospectively supported; prospective validation remains required. DG-7: Conceptually defined as a recurring lifecycle review gate; longitudinal validation remains required.

## Data Availability

The raw data supporting the conclusions of this article will be made available by the authors upon request.
